# The stringent response regulates the poly-β-hydroxybutyrate (PHB) synthesis in *Azotobacter vinelandii*

**DOI:** 10.1371/journal.pone.0299640

**Published:** 2024-04-04

**Authors:** Cristian Camilo Ortiz-Vasco, Soledad Moreno, Luis Alonso Quintero-Navarro, Juliana Berenice Rojo-Rodríguez, Guadalupe Espín

**Affiliations:** 1 Departamento de Microbiología Molecular, Instituto de Biotecnología, Universidad Nacional Autónoma de México (UNAM), Cuernavaca, Morelos, México; 2 Facultad de Ciencias, Universidad Nacional Autónoma de México (UNAM), Ciudad de México, México; CSIR-Institute of Himalayan Bioresource Technology, INDIA

## Abstract

The stringent response exerted by (p)ppGpp and RNA-polymerase binding protein DksA regulates gene expression in diverse bacterial species. To control gene expression (p)ppGpp, synthesized by enzymes RelA and SpoT, interacts with two sites within the RNA polymerase; site 1, located in the interphase between subunits β’ and ω (*rpoZ*), and site 2 located in the secondary channel that is dependent on DksA protein. In *Escherichia coli*, inactivation of *dksA* results in a reduced sigma factor RpoS expression. In *Azotobacter vinelandii* the synthesis of polyhydroxybutyrate (PHB) is under RpoS regulation. In this study, we found that the inactivation of *relA* or *dksA*, but not *rpoZ*, resulted in a negative effect on PHB synthesis. We also found that the *dksA*, but not the *relA* mutation reduced both *rpoS* transcription and RpoS protein levels, implying that (p)ppGpp and DksA control PHB synthesis through different mechanisms. Interestingly, despite expressing *rpoS* from a constitutive promoter in the *dksA* mutant, PHB synthesis was not restored to wild type levels. A transcriptomic analysis in the *dksA* mutant, revealed downregulation of genes encoding enzymes needed for the synthesis of acetyl-CoA, the precursor substrate for PHB synthesis. Together, these data indicate that DksA is required for optimal expression of RpoS which in turn activates transcription of genes for PHB synthesis. Additionally, DksA is required for optimal transcription of genes responsible for the synthesis of precursors for PHB synthesis.

## Introduction

The stringent response is a ubiquitous bacterial reaction triggered by nutrient deprivation and mediated by the intracellular concentrations of ppGpp and pppGpp. These secondary messengers, jointly referred to as (p)ppGpp, control gene transcription, mRNA translation, and protein activity to adjust the metabolism and growth rate to environmental conditions [1]. The intracellular levels of (p)ppGpp are the result of two enzyme activities called RelA and SpoT. These enzymes have a synthesis and synthesis/degradation activity, respectively. In some bacteria, *relA/spoT* mutants (pppGpp^0^), showed defective growth in minimal medium [2].

The main mechanism for (p)ppGpp control on gene expression in most bacteria involves its binding to RNA polymerase at two specific sites: a site 1 on the interphase between β’ and ω (*rpoZ*) subunits, and a site 2 on the secondary channel, which is dependent on the DksA protein. Thus, the inactivation of *rpoZ* (ω subunit) and *dksA* genes removes the (p)ppGpp binding sites 1 and 2, respectively [3].

The presence of site 2 on RNA polymerase allowed the understanding of the *in vitro* and *in vivo* differences in gene expression [4]. Some studies indicated that site 2 is important for transcription activation and modulates the response to (p)ppGpp concentrations. On the other hand, the only presence of site 1 is enough for the repression of gene expression [3].

An RNAseq analysis in a strain that synthesized (p)ppGpp by constitutive expression of *relA*, revealed that the major effect on whole gene expression was due to (p)ppGpp binding to the RNA polymerase, rather than binding to other proteins [5].

In some bacteria, the expression of the alternative sigma factor RpoS is under the control of the DksA and (p)ppGpp; in *Escherichia coli*, *Salmonella typhimurium*, and *Vibrio cholerae* DksA regulates RpoS at a transcriptional and translational level. In *E*. *coli*, the activity of *rpoS* promoters was diminished in a *dksA* mutant; furthermore, an increase in the levels of (p)ppGpp did not affect the *rpoS* transcription in this mutant [6]. In *Salmonella typhimurium* a *dksA* mutant, but not a *relA/spoT* mutant, has reduced levels of RpoS [7]; and in *Vibrio cholerae*, the expression of RpoS in a *dksA* mutant was diminished at transcriptional and translational levels [8].

*Azotobacter vinelandii* is a soil bacterium belonging to the *Pseudomonadaceae* family that produces poly-β-hydroxybutyrate (PHB), a biopolymer of industrial relevance. In this bacterium, the enzymes for the synthesis of PHB are encoded in the *phbBAC* operon [9]. Transcription of this operon initiates from two promoters: pB1 activated by the transcriptional activator PhbR, and pB2, an RpoS dependent promoter. On the other hand, the transcription of *phbR* is also dependent on RpoS; thus, the inactivation of *rpoS* or *phbR* significantly reduced the synthesis of PHB [10, 11].

In *A*. *vinelandii*, the synthesis of PHB starts with the condensation of two molecules of acetyl-CoA by the β-ketothiolase PhbA, to produce acetoacetyl-CoA, which is reduced by the NADPH-dependent acetoacetyl-CoA reductase PhbB, producing the β-hydroxybutyryl-CoA monomer which is polymerized by the PHB synthase PhbC. [9]. The synthesis of PHB is closely related to the functioning of the TCA cycle, since unbalanced growth conditions, such as an excess of a carbon source and other nutrient is growth-limiting, or by mutations that cause a slow-down in the TCA activity lead to a higher availability of acetyl-CoA for the synthesis of PHB [12, 13].

In this study, we investigated the regulatory roles of the stringent response in the control of PHB synthesis in *A*. *vinelandii*. Our results indicate that an effect of a *dksA* mutation on PHB synthesis is a downregulation of the expression of *rpoS*. We also found that some carbon metabolism genes necessary for the synthesis of acetyl-CoA the substrate for PHB synthesis are downregulated in the *dksA* mutant. Additionally, in the *relA* mutant, the reduction of PHB synthesis was not due to a reduction in *rpoS* expression.

## Results

### Inactivation of *relA* or *relA/spoT* genes reduces PHB accumulation

A search in the genome of *A*. *vinelandii* identified *avin37060* and *avin02810* genes encoding proteins sharing a 48% and 54% identity with the RelA and SpoT proteins from *E*. *coli*. Sequences analysis of these proteins showed the presence of domains characteristic of (Rel/Spo homolog) RSH proteins, implying that *avin37060* encodes a RelA type (p)ppGpp synthase while *avin02810* encode a SpoT protein with (p)ppGpp synthase and hydrolase activities.

**Fig 1:**
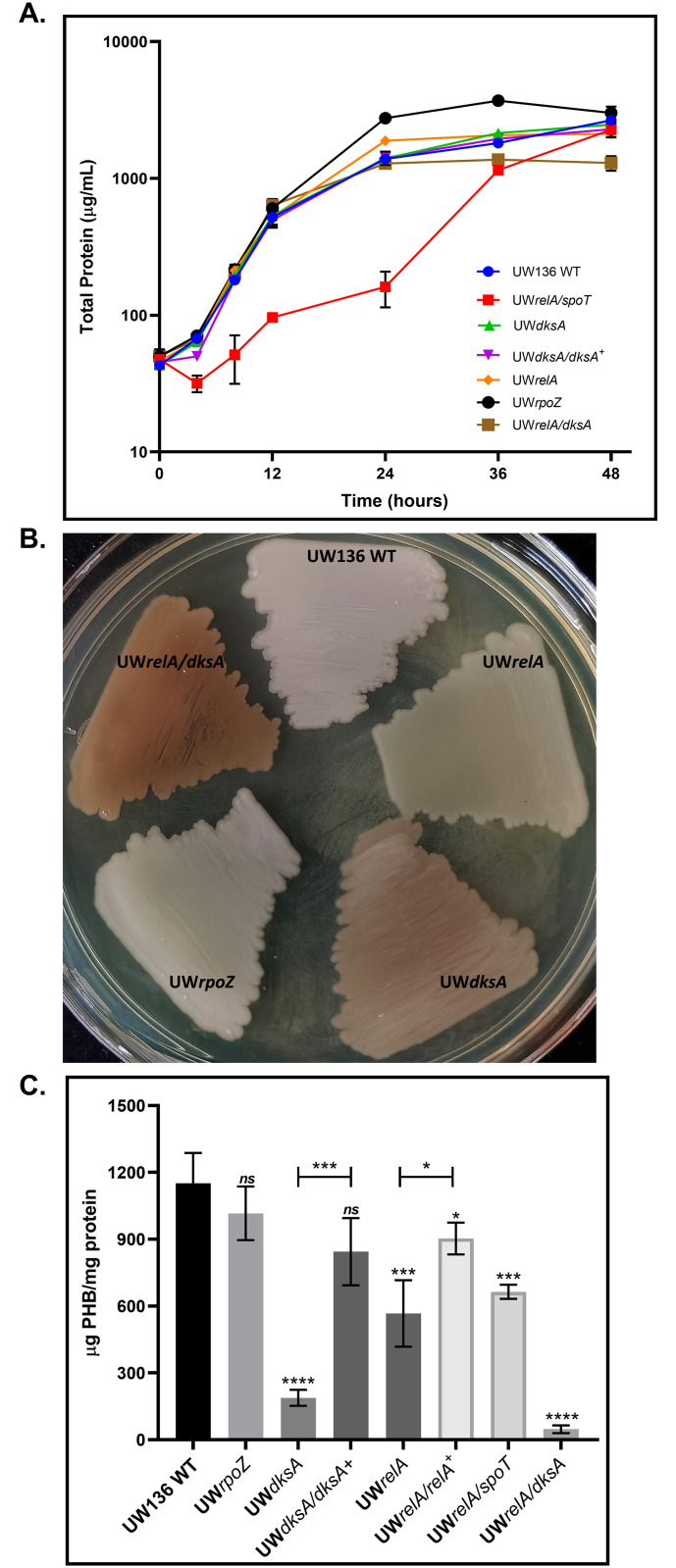
The stringent response regulates the PHB accumulation. (A), Growth kinetics of *A*. *vinelandii* strains in liquid PY medium at 30°C. (B) PHB synthesis phenotype of strains grown in PY solid medium, and (C), PHB quantification in PY liquid medium for 36 hours. The white color indicates PHB accumulation. A less white appearance means reduction or no accumulation of PHB. The mean from three independent experiments is presented. Statistical significance is t student p<0.05 with respect to wild type. Statistical significance is t student p<0.05.

To elucidate the involvement of (p)ppGpp in PHB synthesis, we constructed UW136 derivative strains carrying a *relA*::Km mutation and a double mutant *relA*::Km/*spoT*::Gm to obtain UW*relA* and UW*relA/spoT* strains, which as shown in Fig 1B are less white than UW136. This phenotype is due to a decreased PHB accumulation. Fig 1C shows quantification of PHB in these mutants, where a reduction in PHB accumulation of 50% in the UW*relA*, and 56% in the UW*relA/spoT* strains was observed compared with the wild type. As depicted in Fig 1A, the inactivation of *relA/spoT* genes negatively affected growth. A complemented UW*relA*/*relA*^+^ strain was also constructed. As expected, the synthesis of PHB in the complemented strain increased to 77% of the wild type (Fig 1C).

### The inactivation of *dksA* but not of *rpoZ* diminishes PHB synthesis in *A*. *vinelandii*

As mentioned above, the main mechanism though which (p)ppGpp exerts its effects involves binding to RNA polymerase at two distinct sites, one involving the ω (*rpoZ*) subunit and the other requiring the presence of the DksA protein. We identified in the *A*. *vinelandii* genome a *rpoZ* homolog (*avin02820*) and a *dksA* homolog (*avin42640*).

To determine whether the removal of (p)ppGpp binding sites in RNA polymerase affects PHB synthesis, we constructed strains UW*dksA* and UW*rpoZ* each carrying non-polar *dksA*::Sp and *rpoZ*::Gm mutations, as *rpoZ* and *spoT* are transcribed as an operon (S1 Fig). Fig 1A shows that the inactivation of *dksA* or *rpoZ* had no effect on growth. However, in the absence of *dksA*, PHB synthesis was reduced by 84% compared to the wild type, whereas PHB synthesis in the UW*rpoZ* mutant was not affected (Fig 1B and 1C).

To confirm that the PHB defective phenotype of the UW*dksA* strain was caused by the *dksA* inactivation, we constructed a complemented UW*dksA*/*dksA*^+^ strain. As shown in Fig 1C, PHB synthesis in the complemented strain increased to 72% of the wild type.

### PHB synthesis is abolished in the UW*relA/dksA* double mutant strain

As shown above, the *relA* mutation reduced PHB synthesis by 50%, while in the *dksA* mutant, the synthesis of this polymer was reduced by 86%. These results suggested the possibility that the effect of DksA is independent of (p)ppGpp, or that DksA exerts its control through two distinct pathways, one of which may involve (p)ppGpp binding.

To investigate further, we constructed a double mutant UW*relA/dksA*, and assessed its PHB phenotype. As shown in Fig 1C, PHB production in this strain was abolished. This result agrees with the hypothesis that (p)ppGpp and the DksA protein regulate PHB synthesis through separate and independent mechanisms, implying that, (p)ppGpp levels, provided by RelA, exert control over PHB synthesis via an alternative pathway that does not involve binding to DksA-related site on RNA polymerase. Moreover, the lack of effect of the *rpoZ* mutation on PHB synthesis suggests that the (p)ppGpp effect on PHB is unrelated to its binding to site 1 in the polymerase.

Strains UW*dksA* and UW*relA/dksA* exhibited a light brown color compared to the white color observed in the wild type strain (Fig 1B). The cause of this phenotype and if it is related to PHB is not known, but it may be due to the production of a pigment caused by the pleiotropic nature of the *dksA* mutation.

### RpoS expression is reduced in the UW*dksA* mutant strain

**Fig 2:**
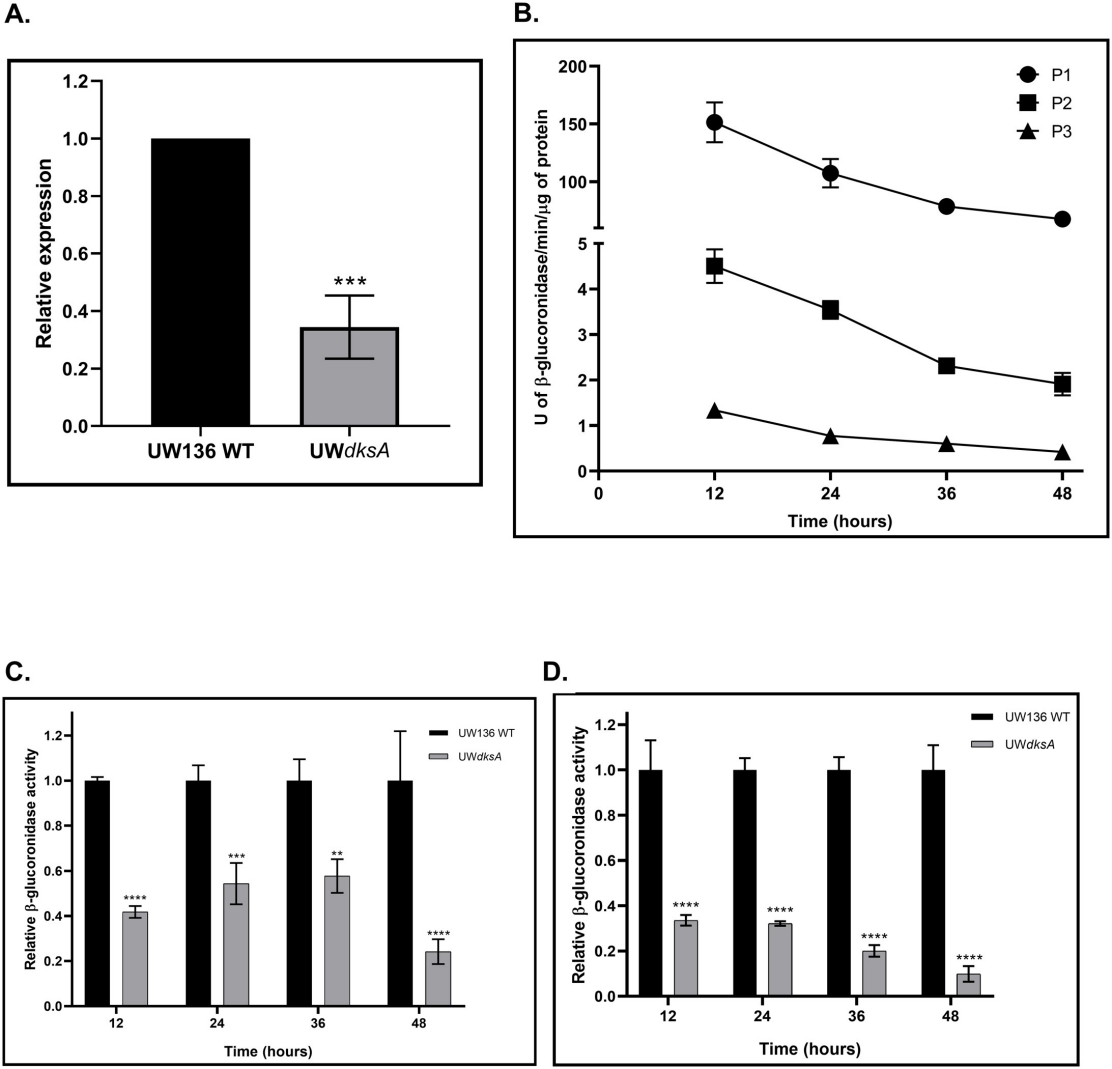
The *dksA* mutation reduces *rpoS* expression and its promoter activity. (A). Relative expression of *rpoS* gene determined by RT-qPCR in the UW*dksA* mutant and the wild type strain after 36 hours incubation on PY medium. (B). β-glucuronidase activity in the wild type strain UW136 carrying transcriptional *rpoS*::*gusA* fusions to the P1, P2, and P3 promoters. (C). Relative β-glucuronidase activity of the transcriptional fusion that contains *rpoS*::*gusA* P1 promoter in the UW*dksA* mutant with respect to wild type. (D). Relative β-glucuronidase activity of transcriptional fusion that contains all promoters (*rpoS*::*gusA*AP) in the UW*dksA* mutant with respect to wild type. The results are the mean from three independent experiments. Statistical significance is t student p<0.05.

DksA regulates the expression of RpoS in several bacterial species. Thus, we investigated whether the dksA mutation in the UW*dksA* resulted in diminished expression of RpoS, required for PHB synthesis. We employed RT-qPCR, to quantify the effect of the *dksA* mutation on *rpoS* transcription. Fig 2A shows that the *rpoS* transcript level in the UW*dksA* mutant was reduced by 70%.

A previous study [14], and an analysis carried out here using *Softberry software*, indicated that *rpoS* transcription in *A*. *vinelandii* initiates from three promoters; P1, P2, and P3. We constructed UW136 derivative strains carrying the *rpoS*::*gusA* transcriptional fusion with all 3 promoters (*rpoS*::*gusA*AP), as well as with only P1, P2, or P3 promoters (Fig 2B and S2 Fig). β–glucuronidase activity measurements in these strains (Fig 2B) indicates that the P1 promoter accounted for about 95% of *rpoS* transcriptional activity, with P2 and P3 jointly contributing a 5% in the wild type strain.

To confirm the positive effect of DksA on *rpoS* transcription we also constructed UW*dksA* mutant derivatives, containing the *rpoS*::*gusA* fusion with P1 or all promoters (*rpoS*::*gusA*AP). Fig 2C, shows that *rpoS* transcription from the P1 promoter was reduced by 30–60% in the *dksA* mutant. Interestingly, a significant reduction was observed in the strain carrying the fusion with all 3 promoters (Fig 2D), indicating a positive role of DksA in *rpoS* transcription.

### The *dksA* mutation, but not the *relA* mutation, reduces RpoS protein levels without affecting stability

**Fig 3:**
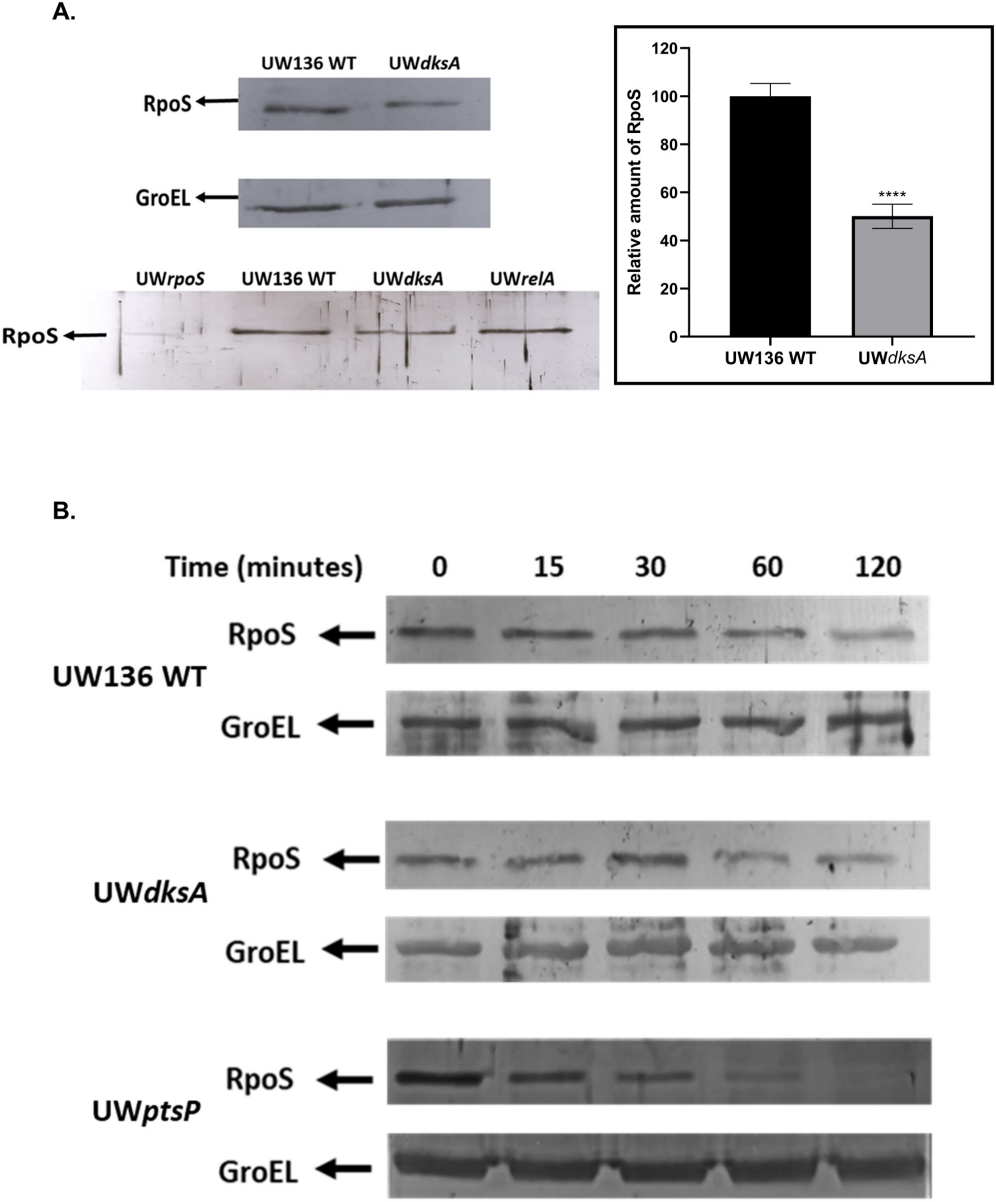
Protein levels of RpoS were reduced in the UW*dksA* mutant, but its stability was not affected. (A). Western blot assay and relative amount of RpoS was determined in UW*dksA* and UW*relA* mutants after 36 hours of incubation on PY medium using polyclonal anti-RpoS antiserum. The relative amount of RpoS in the UW*dksA* was determined by ImageJ software. (B). RpoS Protein stability was determined after adding kanamycin (0.5mg/mL) to block protein synthesis and was compared to the wild type and a UW*ptsP* mutant with a shorter half-life [15].

In *Escherichia coli*, the *dksA* mutation was reported to affect both *rpoS* transcription and RpoS protein levels even under conditions of (p)ppGpp overproduction [6]. We determined the effect of the *dksA* and *relA* mutations on RpoS level and stability. Western blot assays revealed that in line with reduced *rpoS* transcription, RpoS protein levels in the UW*dksA* mutant were diminished by about 50% compared to those in the wild type strain, whereas RpoS levels in the UW*relA* mutant were not affected (Fig 3A).

We also assessed the *in vivo* stability of RpoS. As shown in Fig 3B, the RpoS protein in the UW*dksA* mutant exhibited a half-life similar to that observed in the wild type. As a control, we employed UW*ptsP* strain, where RpoS half-life was previously shown to be reduced [15]. The negative effect on RpoS levels in the UW*dksA* but not in UW*relA* strain, further support the proposal that (p)ppGpp levels and DksA protein affect PHB synthesis through different pathways.

### Transcription of *rpoS* from a constitutive promoter in the UW*dksA* mutant does not restore PHB synthesis

**Fig 4:**
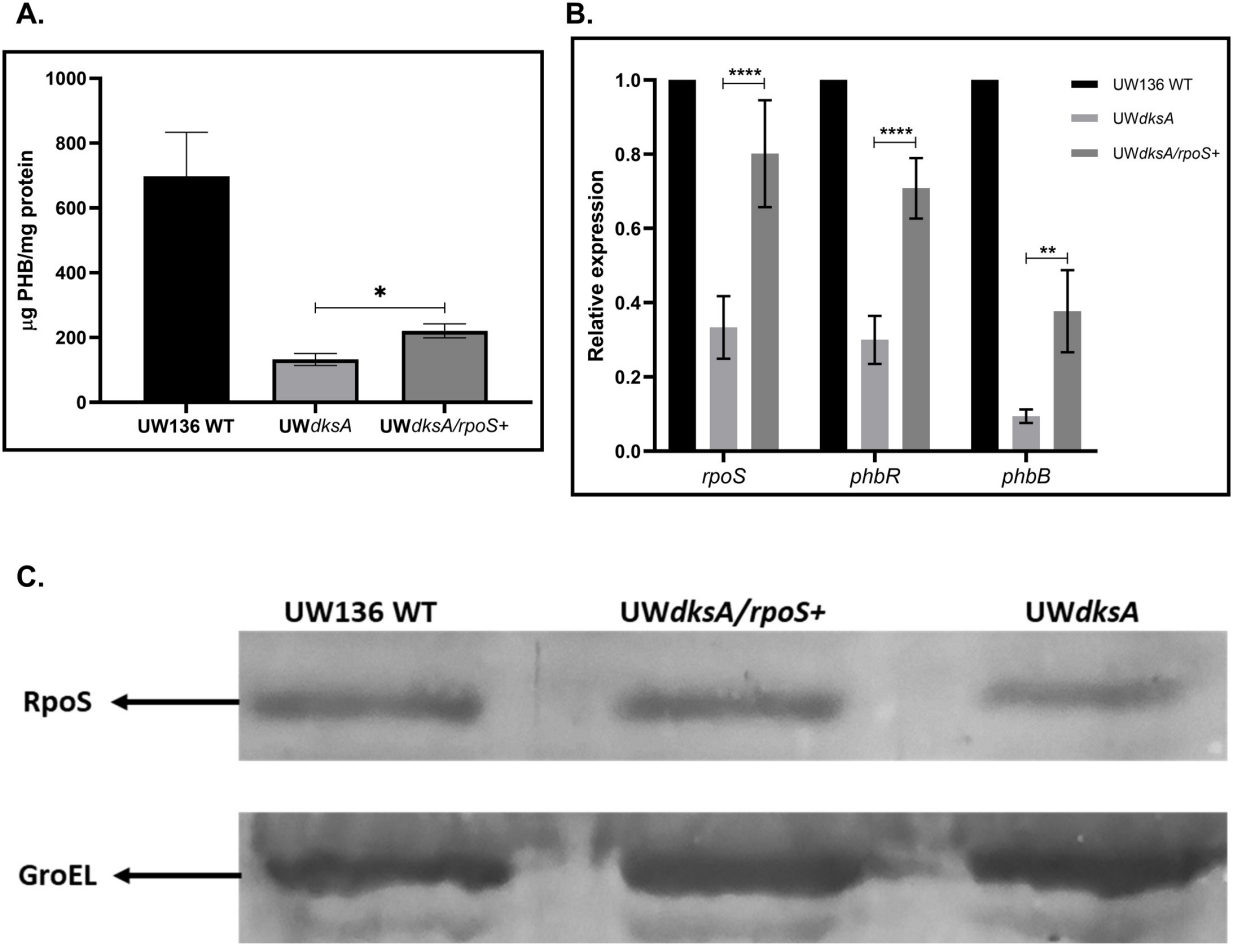
Complementation with a *rpoS* copy from a constitutive promoter increased the *rpoS*, *phbR*, and *phbB* transcription and the RpoS protein but did not restore the PHB production on UW*dksA* mutant. (A). Quantification of PHB production. (B). Relative expression of *rpoS*, *phbR*, and *phbB* genes determined by RT-qPCR in UW*dksA* and UW*dksA/rpoS+* strains. (C). Detection of RpoS by Western blot assay. Determination of transcript levels and RpoS protein were carried out after 36 hours of incubation in a PY liquid medium. The results are the mean from three independent experiments. Statistical significance is t student p<0.05.

To evaluate whether the reduced PHB accumulation in the UW*dksA* mutant primarily resulted from diminished *rpoS* expression, we constructed UW*dksA*/*rpoS*^+^ strain, containing a *rpoS* gene expressed from the *gyrA* constitutive promoter. The expectation was that by increasing *rpoS* transcription in a DksA-independent manner, the PHB-negative phenotype caused by the *dksA* mutation would be restored. As shown in Fig 4B, the transcript level of *rpoS* determined by RT-qPCR, along with the RpoS protein level (Fig 4C) were significantly increased. As expected an increased expression of *phbR* and *phbB* was also observed in the UW*dksA*/*rpoS*^+^ strain (Fig 4B). However, PHB synthesis was only partially restored corresponding to 25% of the wild type strain (Fig 4A).

These results suggest that the DksA protein exerts an additional level of control over PHB synthesis, that is independent of its regulation of RpoS expression.

### Genes encoding enzymes for PHB precursors synthesis are downregulated in the UW*dksA* strain

**Fig 5:**
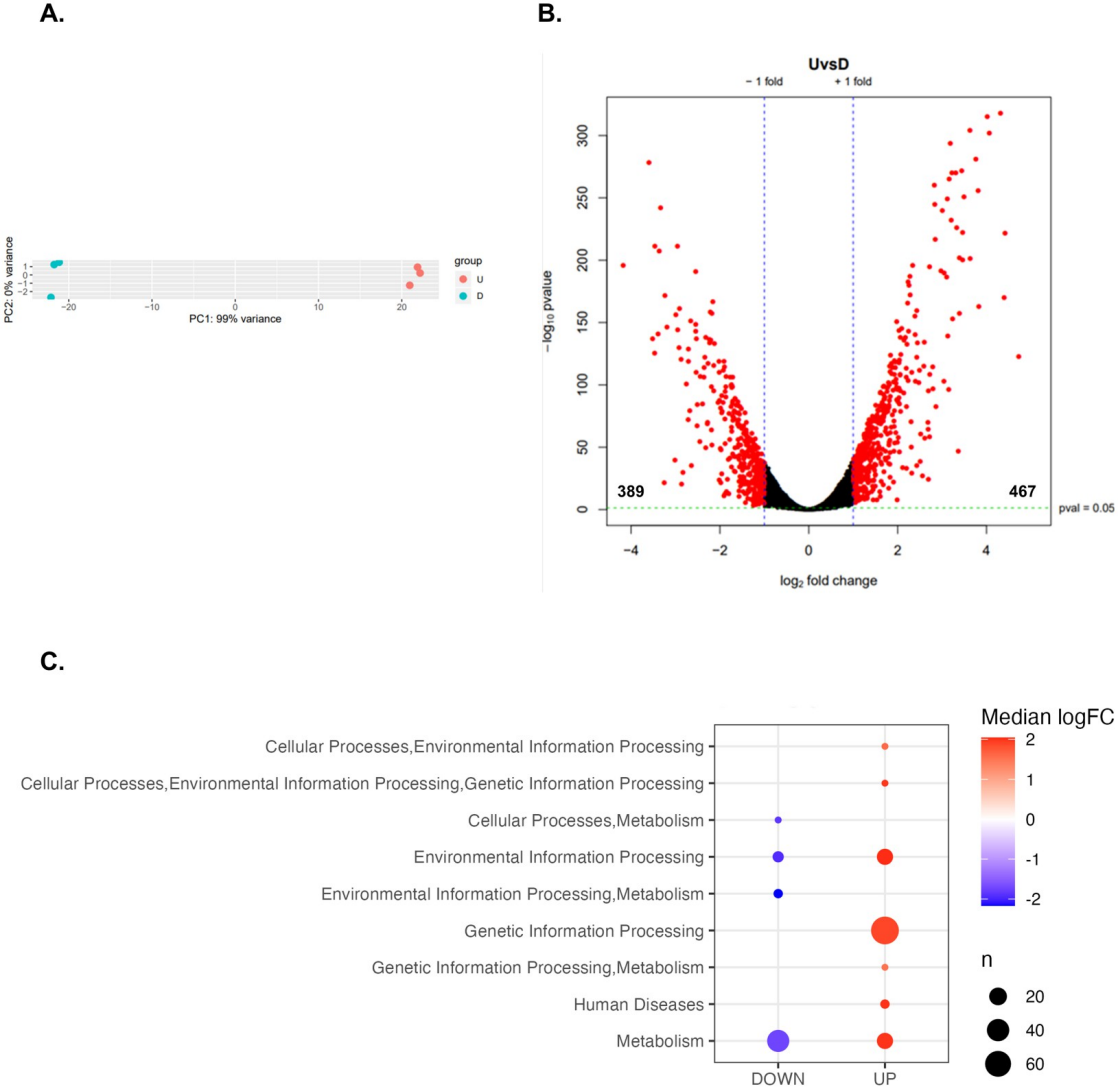
UW*dksA* mutant transcriptomic profile. (A). Principal component analysis identified clustering of UW*dksA* mutant (blue) with respect to UW136 (red). (B). Differential gene expression analysis identified 856 UW*dksA* mutant DEGs compared to UW136. (log_2_ FC ±1 cut off, pValue <0.05). (C). Clustering of the enriched Biological Process GO: terms. Spots represent GO: terms, one gene can belong to more than one GO: term. Spot size relates the number of genes in each term and the color indicates the enrichment significance.

To identify an RpoS-independent and alternative pathway throughout DksA coregulates PHB synthesis, we conducted a transcriptome analysis comparing the UW*dksA* mutant to the wild type. Principal component analysis (PCA) highlighted distinct clustering of replicate strains, with the most significant variance (99%) between UW*dksA* and UW136 strains being captured by the first principal component (PC1) (Fig 5A).

Differential gene expression analysis as performed using IDEAMEX software revealed 856 differentially expressed genes (DEGs) between UW*dksA* and UW136 strains, comprising 467 upregulated genes and 389 downregulated genes (Fig 5B, log_2_ FC ±1, pValue<0.05). Gene list enrichment analysis of the UW*dksA* mutant identified enrichment terms in Metabolism, Genetic Information Processing, and Environmental Information Processing (Fig 5C). Clustering of enriched Metabolism and Genetic Information Processing GO term clusters showed cased prominence in “metabolic pathways”, “pyruvate metabolism”, “biosynthesis of secondary metabolism” and “ribosome” (S3 Fig).

**Fig 6:**
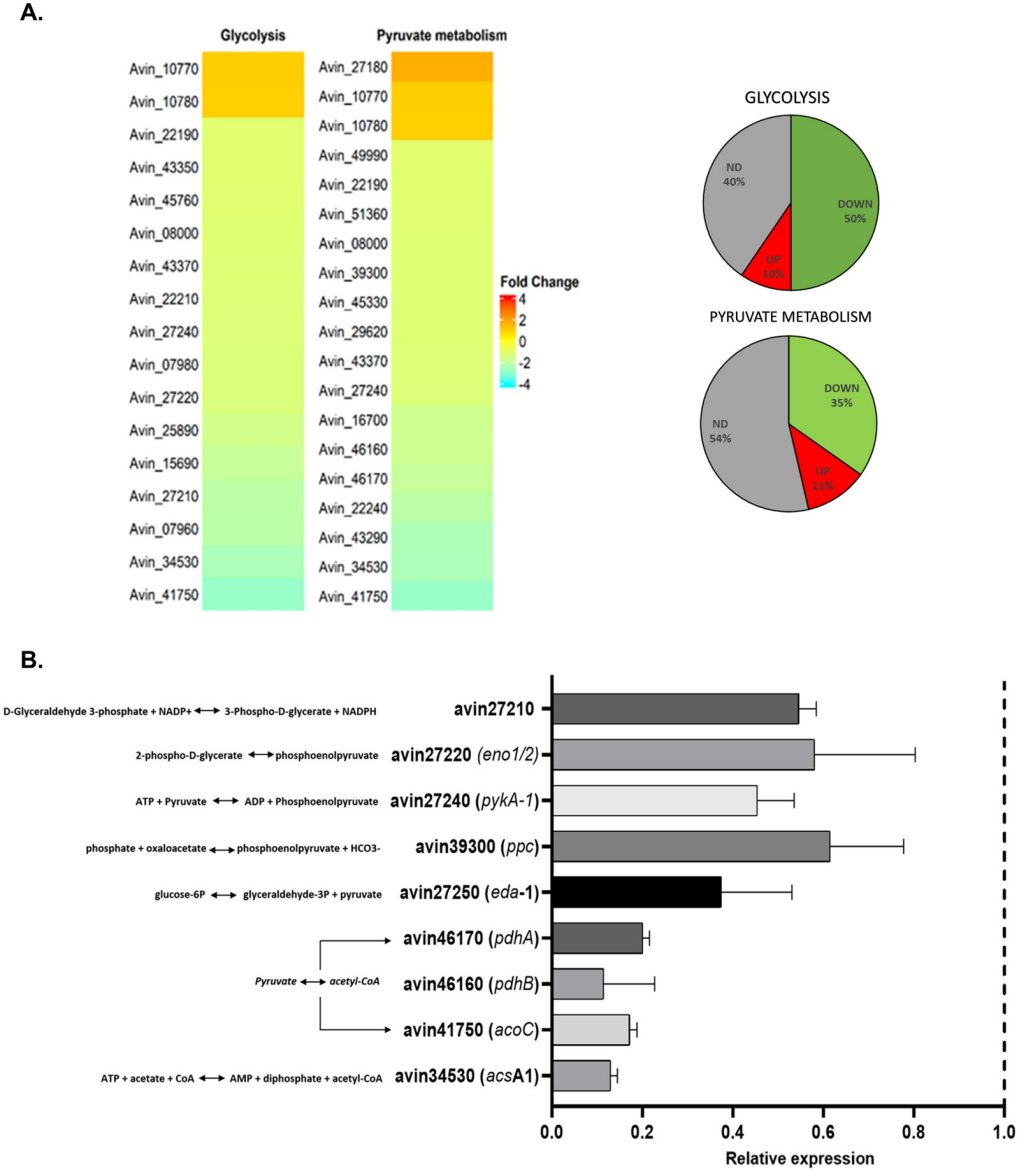
Genes that code enzymes necessary for the synthesis PHB precursors were downregulated in the UW*dksA* mutant. (A). Glycolysis and pyruvate metabolism pathways deposited in KEEG to *A*. *vinelandii* are represented. The color scale indicated the differential regulated between UW*dksA* mutant and UW136 (log_2_ FC ±1 pValue <0.05). Right. Percent of genes in the metabolic pathway that change the expression in the UW*dksA* mutant respect to the wild type (ND: No differential expression). (B). RT-qPCR of genes related to phosphoenolpyruvate, pyruvate, and acetyl-CoA synthesis from UW*dksA* mutant compared to the UW136.The results are the mean from three independent experiments. Statistical significance is t student p<0.05.

We explored metabolic pathways responsible for the synthesis of precursors for PHB synthesis. As shown in Fig 6A and S3 Fig, the glycolysis and pyruvate metabolism pathways were downregulated in the UW*dksA* strain. We validated the reduced expression of nine genes encoding enzymes required for PHB precursors (phosphoenolpyruvate, pyruvate, and acetyl-CoA) via RT-qPCR (Fig 6B). These findings suggest that the downregulation of these genes may contribute to the observed negative impact on PHB synthesis in the UW*dksA* strain.

Additionally, other enriched GO term clusters upregulated in the UW*dksA* mutant pertained to Genetic Information Processing, particularly “aminoacyl tRNA biosynthesis” and “ribosome” (S3 Fig, log_2_ FC ±1, pValue <0.05). This indicates that the *dksA* mutation affects the transcription of genes associated to ribosomal proteins, implying a repressive role of DksA on genes linked to ribosome biogenesis. in *A*. *vinelandii*

## Discussion

Recent research has shed light on the broad implication of the stringent response in bacterial physiology, extending its influence beyond amino acid deprivation to encompass transcriptional regulation of hundreds of genes [2]. The (p)ppGpp synthesized by RelA/SpoT proteins, and the DksA protein are required to carry out the changes in transcription observed during the stringent response. The impact of (p)ppGpp levels in the synthesis of polyhydroxyalkanoates (PHAs) including PHB, in some bacteria has been investigated. In model organisms like *Cupriavidus necator* H16 (previously called *Ralstonia eutropha*), mutations in *relA/spoT* genes have shown to regulate PHB synthesis and degradation [16, 17]. Similar effects have been observed in species such as *Pseudomonas putida* KT2440, *Pseudomonas chlororaphis* PA23, and *Rhizobium etli* upon inactivation of *relA/spoT* [18–20].

This study aimed to elucidate the relevance of stringent response genes in PHB synthesis in *A*. *vinelandii*. Like another γ-proteobacteria *A*. *vinelandii* possesses homologs of the RelA and SpoT proteins. Our construction of a UW*relA* single mutant and a UW*relA/spoT* double mutant generating a (p)ppGpp^0^ strain revealed that as in other PHAs-producer bacteria, *A*. *vinelandii relA/spoT* mutations led to a reduction in PHB synthesis.

(p)ppGpp primarily exert its regulatory role though binding to RNA polymerase at two distinct sites: site 1, dependent on the ω subunit (*rpoZ*), and site 2, dependent on the RNA polymerase binding protein DksA [2]. This study showed that mutational inactivation of *rpoZ* had no effect on PHB synthesis. In contrast, the UW*dksA* mutant exhibited a more pronounced reduction in PHB synthesis than the UW*relA* mutant. Interestingly, UW*relA/dksA* double mutant showed accumulative effect in agreement with the notion that (p)ppGpp and DksA independently regulate PHB synthesis through different pathways.

It is noteworthy that in *E*. *coli* many phenotypes regulated by stringent response are potentiated by the (p)ppGpp-DksA interaction [21]. However, DksA can also act independently of (p)ppGpp to modulate RNA polymerase activity. Negative and positive regulation by (p)ppGpp has been studied and found that either site 1 or site 2 alone are sufficient for the negative regulation of promoter activity, while site 2 is necessary for positive regulation, with the presence of both sites enhancing activity [5].

Our results agree with the positive role of the DksA protein in PHB synthesis, even in conditions where normal (p)ppGpp levels are present, as in the UW*dksA* mutant. This suggest that DksA, independently of (p)ppGpp, promotes the expression of genes required for the PHB synthesis.

In *A*. *vinelandii*, the sigma factor RpoS has been shown to activate transcription of *phbR* and the PHB biosynthetic operon *phbBAC* [10, 11]. In some bacteria like *E*. *coli* and *Borrelia burgdorferi*, a *dksA* mutant exhibited undetectable RpoS level under various conditions, even in the presence of high (p)ppGpp levels [6, 22]. In *Vibrio cholerae* DksA was found to regulate RpoS expression at the transcriptional and translational level [8].

This study showed that in *A*. *vinelandii*, transcription of *rpoS* is initiated from three promoters, with P1 contributing with 95% of *rpoS* transcriptional activity. Interestingly, the UW*dksA* mutant, showed a significant reduction in transcription from the P1 promoter. Moreover, in the UW*dksA* strain with the *rpoS*::*gusAAP* transcriptional fusion, the reduction of β-glucuronidase activity was higher than in the strain with only the P1 promoter, suggesting the presence of regulatory elements in the DNA region downstream P1. In concordance with the decreased *rpoS* transcription, expression of *phbBAC* and *phbR* was significantly reduced in the UW*dksA* mutant. Taken together these results indicated the necessity of DksA for optimal RpoS expression, which in turn activates transcription of PHB genes.

As a result of the reduction in the *rpoS* transcription, the RpoS protein levels were also lowered in the UW*dksA* mutant, while the UW*relA* mutant exhibited wild type RpoS levels. This indicated that, (p)ppGpp regulates PHB production through an RpoS-independent pathway.

To ascertain whether the reduction of *rpoS* transcription observed in the UW*dksA* mutant was responsible for the reduction in PHB synthesis, we investigated if DksA independent expression of *rpoS*, could restore the PHB synthesis. As expected, in the UW*dksA* mutant with the *rpoS* gene transcribed from a *gyrA* promoter, the RpoS protein levels were restored to those of the wild type. However, PHB synthesis was only partially restored, suggesting that DksA also controls PHB synthesis through an RpoS-independent pathway.

To further investigate the regulatory mechanisms by which DksA controls PHB synthesis independently of RpoS, we carried out a transcriptome analysis comparing the UW*dksA* mutant with the wild type UW136. The RNA-seq results showed significant transcriptional changes, in genes involved in metabolism, metabolic pathways, pyruvate metabolism, and biosynthesis of secondary metabolism, which were downregulated. Additionally, genes related to Genetic Information Processing such as aminoacyl tRNA biosynthesis and ribosome-related genes were upregulated.

Some of the downregulated genes involved in carbon metabolism encoded enzymes responsible for the synthesis of phosphoenolpyruvate, pyruvate, and acetyl-CoA, which are precursors for PHB synthesis. This suggest that the decrease in the level of these enzymes, may contribute to the observed decrease in PHB synthesis in the UW*dksA* mutant, as well as in its derivative strain expressing *rpoS* from a constitutive promoter. No negative effect on growth was observed in the UW*dksA* mutant despite the decreased expression of the carbon metabolism genes. This may be due to a lack of effect of the *dksA* mutation in exponentially growing cells, as downregulation of these genes was observed in cell in stationary phase where the synthesis of PHB is carried out.

As in *A*. *vinelandii*, in other bacteria, carbon metabolism enzymes of the glycolytic pathway and the tricarboxylic acid (TCA) cycle have been reported as part of the DksA and (p)ppGpp regulon [23]. Also, in the *Enterobacteriaceae*, *Yersinia enterocolitica*, the response to DksA and RelA/SpoT proteins has been studied, revealing the downregulation of genes encoding enzymes of the TCA cycle [24].

In *E*. *coli*, DksA is also involved in the response to glucose-phosphate stress, which leads to growth inhibition due to the accumulation of sugar phosphates, or to depletion of glycolytic intermediates. The small RNA SgrS and its transcriptional activator SgrR alleviate this stress in part by repressing sugar phosphates transport [25]. This mechanism potentially present in *A*. *vinelandii* remains to be explored. The presence of small RNAs regulated by the stringent response in *A*. *vinelandii* is an attractive possibility to study.

In summary, this study along with research carried out in other bacterial species, has established a link between stringent response effectors and carbon metabolic networks. We propose that in *A*. *vinelandii* this link is likely to affect PHB synthesis.

Finally, early studies on the effects of the stringent response demonstrated the inhibition of ribosome biogenesis and transcription of rRNA genes [2]. More recent transcriptomic analysis revealed upregulation of genes coding for ribosomal proteins and ribosome assembly factors in *E*. *coli* and *Yersinia enterocolitica* [5, 24]. This study revealed that DksA functions as a repressor of ribosomal synthesis genes in *A*. *vinelandii*. Notably in *Pseudomonas* species, phylogenetically close related to *A*. *vinelandii*, a *dksA* mutation also affected the transcription of genes linked to ribosomal proteins and ribosome biogenesis [26, 27]. However, whether the upregulation of these genes in *A*. *vinelandii* affects PHB synthesis remains to be investigated.

**Fig 7:**
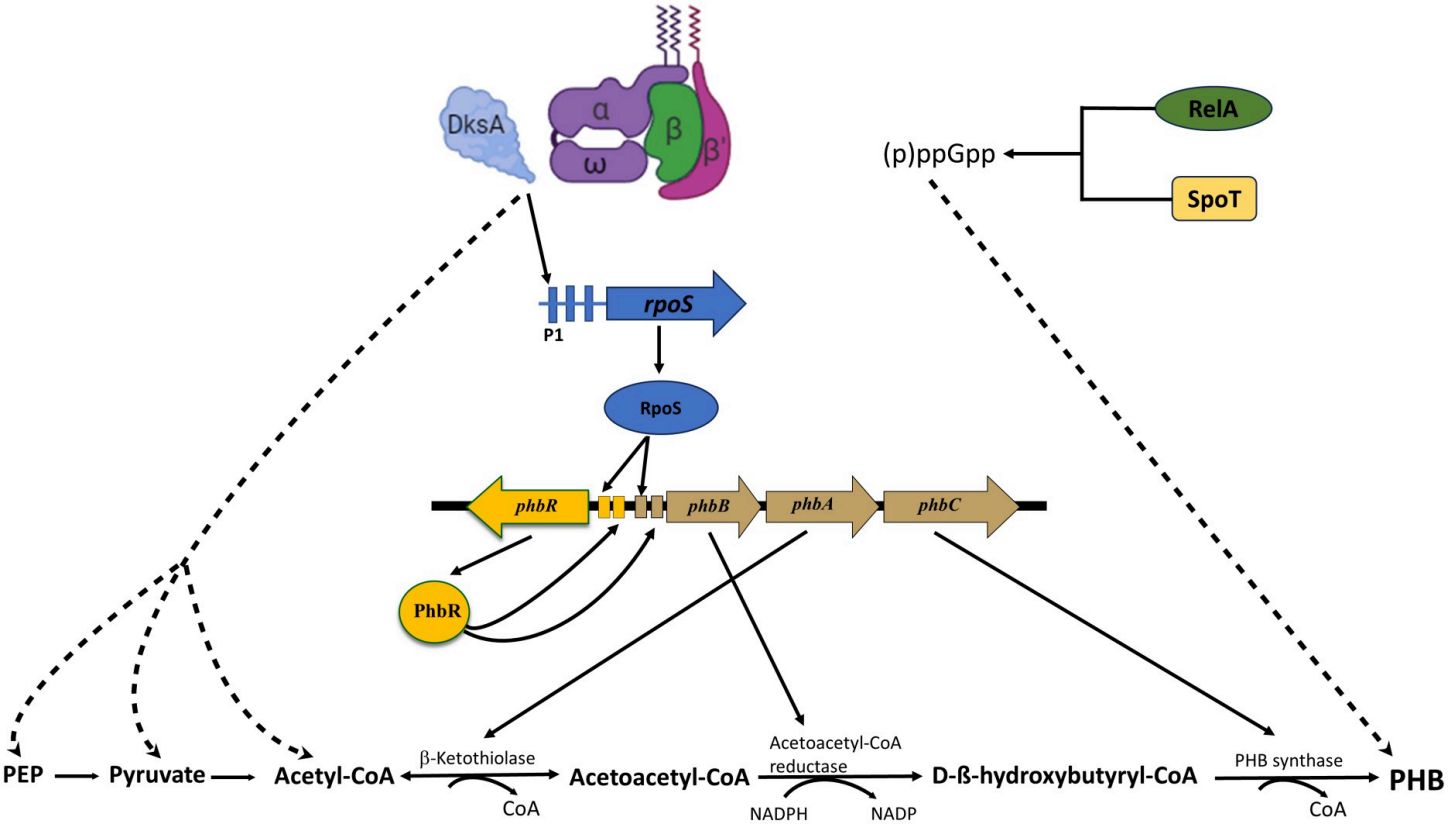
Model for the regulation of PHB synthesis by DksA and (p)ppGpp in *A*.*vinelandii.* Promoters are indicated as small rectangles; positive regulation is indicated with arrows; dashed lines indicate unknown intermediates and mechanism.

In summary, we propose a model (Fig 7) for the regulation of the synthesis of PHB by (p)ppGpp and DksA in *A*. *vinelandii*, where DksA is necessary for optimal transcription of *rpoS*. In turn, RpoS activates transcription of the PHB biosynthetic genes. On the other hand, DksA is also required for optimal transcription of genes encoding enzymes responsible for the synthesis of acetyl-CoA the substrate for the synthesis of PHB. Finally, (p)ppGpp exerts a positive role on synthesis of PHB by and unknown mechanism independent of DksA and RpoZ.

## Materials and methods

### Strains, media, and culture conditions

The bacterial strains and plasmids used in this study are described in Table 1, and the oligonucleotides used in S1 Table. *A*. *vinelandii* strains were grown in peptone yeast medium added with 2% sucrose (PY) and incubated at 30° C. *E*. *coli* strains were cultured in Luria Bertani (LB) medium at 37°C. Antibiotic concentrations used for *A*. *vinelandii* were as follows: kanamycin (km) 3μg/mL, gentamycin (Gm) 1μg/mL, tetracycline (Tc) 30μg/mL, and spectinomycin (Sp) 100μg/mL.

**Table 1:** Strains and plasmids, used in this study.

Strain	Description	Reference
*E*. *coli* DH5 **α**	endA1 hsdR17 supE44 thi-1λ-recA1 gyrA96 relAΔlacU169φ80 (ΔlacZ ΔM15).	[28]
UW136	*A*. *vinelandii* wild type strain	[29]
UW*rpoZ*	UW136 derivative carrying a *rpoZ*::Gm non polar mutation	This study
UW*relA*	UW136 derivative carrying a *relA*::Km mutation	This study
UW*dksA*	UW136 derivative carrying a *dksA*::Sp mutation	This study
UW*relA/spoT*	UW136 derivative carrying a *relA*::Km and *spoT*::Gm mutations	This study
UW*relA/dksA*	UW*relA* carrying a *dksA*::Sp mutation.	This study
UW*dksA/dksA*^+^	UW *dksA* carrying pJETdksAAC-Km cointegrated into the chromosome	This study
UW*relA/relA*^+^	UW*relA* carrying a wild type *relA* gene transcribed from a *gyrA* promoter	This study
UW*rpoS*::*gusA-P1*	UW136 carrying a *rpoS*::*gusA* gene fusion transcribed from the *rpoS* promoter P1	This study
UW*rpoS*::*gusA-P2*	UW136 carrying a *rpoS*::*gusA* gene fusion transcribed from the *rpoS* promoter P2	This study
UW*rpoS*::*gusA-P3*	UW136 carrying a *rpoS*::*gusA* gene fusion transcribed from the *rpoS* promoter P3	This study
UW*rpoS*::*gusA-AP*	UW136 carrying a *rpoS*::*gusA* gene fusion transcribed from the *rpoS* P1, P2 and P3 promoters	This study
UW*dksA/rpoS*::*gusA-P1*	UW*dksA* carrying a *rpoS*::*gusA* gene fusion transcribed from the rpoS promoter P1	This study
UW*dksA/rpoS*::*gusA-P2*	UW*dksA* carrying a *rpoS*::*gusA* gene fusion transcribed from the *rpoS* promoter P2	This study
UW*dksA/rpoS*::*gusA-P3*	UW*dksA* carrying a *rpoS*::*gusA* gene fusion transcribed from the *rpoS* promoter P3	This study
UW*dksA/rpoS*::*gusA-AP*	UW*dksA* carrying a *rpoS*::*gusA* gene fusion transcribed from the *rpoS* P1, P2 and P3 promoters	This study
UW*dksA*/*rpoS*^+^	UW *dksA* derivative carrying an *rpoS* gene transcribed from a *gyrA* promoter.	This study
**Plasmids**		
pJET1.2	pJET1.2 blunt cloning vector.	Thermo Scientific
pBSL97	Plasmid with a Km cassette.	[30]
pBSL98	Plasmid with a Gm cassette.	[30]
pHP45Ω	Plasmid with omega Sp cassette.	[31]
pUMATc	pUC19 containing a 1kb fragment with the *melA* gene and a Tc cassette.	[32]
pUMATcgusAT	Vector with a *gusA* gene for transcriptional fusions.	[15]
pJET-gyrA	pJET1.2 carrying the promoter region of *gyrA*	[31]
pJETspoT	pJET with *spoT* gene.	This study
pJETrel*A*	pJET with locus *relA*.	This study
pJETdks*A*	pJET with *dksA* locus.	This study
pJETrpoZ	pJet with *rpoZ* gene.	This study
pJETspoT::Gm	pJET with *spoT* gene mutation.	This study
pJETrpoZ::Gm	pJET with *rpoZ*::Gm NP mutation.	This study
pJETrelA::*Km*	pJET with the *relA*::Km mutation.	This study
pJETdksA::Sp	pJET with *dksA*::Sp mutation.	This study
pJETdksAC	pJET with a *dksA* gene and its own promoter.	This study
pJETdksAC-Km	pJETdksAC with a Km-cassette.	This study
pJETrelAC	pJET with a *relA* gene	This study
pJETgyrA-relAC	pJET-gyrA with a *relA* gene from pJETrelAC	This study
pUMAgyrA-relA	pUMA plasmid with a *relA* gene transcribed from a *gyrA* promoter.	This study
pJETP1rpoS	pJET carrying a fragment with the *rpoS* P1 promoter	This study
pJETP2rpoS	pJET carrying a fragment with the *rpoS* P2 promoter	This study
pJETP3rpoS	pJET carrying a fragment with the *rpoS* P3 promoter	This study
pJETAPrpoS	pJET carrying a fragment with the *rpoS* P1, P2 and P3 promoters	This study
pUMAP1rpoS::gusA	pUMATc gusAT derivative carrying a *rpoS*::*gusA*P1 transcriptional fusion	This study
pUMAP2rpoS::gusA	pUMATcgusAT carrying a *rpoS*::*gusA*P2 transcriptional fusion	This study
pUMAP3rpoS::gusA	pUMATcgusAT carrying a *rpoS*::*gusA*P3 transcriptional fusion	This study
pUMAAPrpoS::gusA	pUMATcgusAT carrying a *rpoS*::*gusA*AP transcriptional fusion	This study
pJETrpoS	pJET 1.2 with a *rpoS* gene copy.	This study
pJETgyrA-rpoS	pJETgyrA plasmid with *rpoS* gene copy.	This study
pUMAgyrA-rpoS	pUMA plasmid with an *rpoS* gene transcribed from a *gyrA* promoter	This study

### Construction of plasmids and mutant strains

DNA fragments of 2336, 2833, 663, and 435 bp containing the *spoT*, *relA*, *dksA*, or *rpoZ* genes respectively, were amplified using oligonucleotides spoT-Fw, spoT-Rv, relA-Fw, relA-Rv, dksA-Fw, dksA-Rv, rpoZ-Fw, and rpoZ-Rv, respectively. These fragments were cloned into pJET1.2/blunt vector to generate plasmids pJETspoT, pJETrelA pJETdksA and pJETrpoZ. Restriction sites *Apa*I, *Sal*I, inside *spoT* and *rpoZ* respectively were used to insert a Gm cassette obtained from plasmid pBSL98. Km and Sp cassettes from pBSL99 and pHP45W-Km plasmids were inserted into *Sac*I and *Sma*I sites inside *relA* and *dksA* genes. In *A*. *vinelandii*, the insertion of Gm, Km or Sp cassettes into genes with same orientation as the direction of transcription, produces non-polar mutations which allow transcription of downstream genes in the same operon [33]. The resultant plasmids pJETspoT::Gm, pJETrpoZ::Gm, pJETrelA::Km, and pJETdksA::Sp were confirmed to carry the cassettes cloned in the same direction of transcription of the mutated gene. Plasmids pJETrpoZ::Gm, pJETrelA::Km and pJETdksA::Sp were digested with *Sca*I and used to transform *A*. *vinelandii* UW136 strain. Linearization of these plasmids was carried out to avoid plasmid cointegration into the chromosome, due to single recombination events. Transformants UW*rpoZ*, UW*relA*, and UW*dksA* resistant to Gm, Km or Sp respectively were selected from BS plates supplemented with the respective antibiotics. For the construction of UW*relA/spoT* UW*relA/dksA* double mutants, plasmids pJETspoT::Gm and pJETdksA::Sp were used to transform UW*relA* mutant. The presence of the *spoT*::*Gm*, *rpoZ*::Gm, *relA*::Km, and *dksA*::Sp mutations in these transformants was confirmed by PCR (data not shown).

For the construction of complemented UW*dksA*/*dksA*^+^, a fragment containing *dksA* including its own promoter was amplified using oligonucleotides dksAC-Fw and dksAC-Rev, that was cloned into pJET1.2 to generate plasmid pJETdksAC. A Km-cassette was inserted into a *Sca*I site (located within the pJET1.2), of pJETdksAC to produce plasmid pJETdksAC-Km. This plasmid unable to replicate in *A*. *vinelandii* was transformed into UW*dksA* strain. A Km resistant transformant UW*dksA*/*dksA*^+^, that showed a higher PHB accumulation phenotype than its parental UW*dksA* was selected, and confirmed by PCR to carry pJETdksAC-Km that was cointegrated within the *dksA*::Sp gene by a single recombination event.

For construction of strain UW*relA/relA*^+^, a 2273 bp fragment containing the *relA* gene was amplified using oligonucleotides relAC-Fw-XbaI and relAC-Rev-BamHI, cloned into pJET1.2 to produce pJETrelAC. An *Xba*I-*BamH*I fragment from this plasmid, was excised and cloned into pJETgyrA downstream and in the same direction of the p*gyrA* promoter producing plasmid pJETgyrA-relAC. A *Bgl*II fragment containing the p*gyrA-relA* fusion excised from this plasmid, was cloned into PUMA-Tc carrying the *A*. *vinelandii melA* gene that is used as a neutral site to introduce genes or gene fusions into the *A*. *vinelandii* chromosome [17, 34]. The resulting plasmid PUMAgyrA-relA was linearized and transformed into UW*relA* mutant for isolation of UW*relA/relA*^+^ strains, a Tc resistant transformant where the p*gyrA-relA* fusion was integrated into the *melA* gene by a double recombination event.

Construction of strain UW*dksA/rpoS*^+^. **O**ligonucleotides *rpoS-BamH*I Fw and *rpoS-Xba*I Rv were used to amplify a 1022 bp fragment containing the *rpoS* gene that was cloned into the pJET1.2 vector, resulting in plasmid pJET*rpoS*. A *Bam*HI and *Xba*I fragment containing the *rpoS* gene excised from this plasmid was cloned downstream and in the same orientation as the *gyrA* promoter into the pJET*gyrA* vector [17], to generate plasmid pJET*gyrA-rpoS*. A *Bgl*II fragment with the p*gyrA-rpoS* fusion obtained from this plasmid, was inserted into the *BamH*I site of the *A*. *vinelandii melA* gene of vector pUMA*-Tc* [34], producing plasmid pUMA*gyrA-rpoS*. This plasmid was linearized by *Sca*I digestion and transformed into the UW*dksA* strain, to isolate Tc resistant strain UW*dksA/rpoS*^+^, that carries the *rpoS* gene transcribed from the *gyrA* promoter integrated within the *melA* gene.

To the construction of strains carrying transcriptional *gusA* gene fusions. DNA fragments of 171bp, 145bp, and 137bp containing the *rpoS* P1, P2, P3 promoters respectively, were amplified by PCR using the oligonucleotides: rpoS-P1-Fw and rpoS-P1-Rv, rpoS-P2-Fw and rpoS-P2-Rv, rpoS-P3-Fw and rpoS-P3-Rv. Oligos rpoS-P1-Fw and rpoSP3-Rv were used to obtain a 457pb fragment containing the three *rpoS* promoters (*rpoS* AP). Restriction sites for *Xba*I and *Eco*RI were included in all oligonucleotides. These four fragments shown in S2 Fig were cloned into the pJET1.2 vector to produce plasmids pJETP1rpoS, pJETP2rpoS, pJETP3rpoS and pJETAPrpoS respectively. *Xba*I-*Eco*RI fragments from these plasmids were sequenced and cloned into plasmid pUMATc*gusA*T vector [17] generating plasmids pUMAP1rpoS::*gusA*, pUMAP2rpoS::*gusA*, pUMAP3rpoS::*gusA*, and pUMAAPrpoS::*gusA* respectively that were linearized by *Sca*I digestion and transformed into *A*. *vinelandii* UW136. Tc-resistant transformants were selected resulting in strains UW*rpoS*::*gusA*-P1, UW*rpoS*::*gusA*-P2, UW*rpoS*::*gusA*-P3, and UW*rpoS*::*gusA-*AP. Similarly, these plasmids were used to transform the UW*dksA* strain to generate strains UW*dksA/rpoS*::*gusA*P1, UW*dksA/rpoS*::*gusA*P2, UW*dksA/rpoS*::*gusA*P3, and UW*dksA/rpoS*::*gusA*AP. The integration of the *rpoS*::*gusA* transcriptional fusions by a double recombination event was confirmed by PCR.

### Plasmid transformation

Plasmid transformation was carried out as previously described [34]. Briefly *A*. *vinelandii* cells were grown overnight in Burk’s medium modified by the omission of the Fe and Mo salts. Aliquots of cells were mixed with plasmid DNA on plates of Burk’s medium and incubated overnight at 30 C. The transformants were plated on selective Burk’s medium with the corresponding antibiotic.

### β-glucuronidase activity

The β-glucuronidase activity was determined as described [35], 1 U corresponds to 1 nmol of p-nitrophenyl-β-D-glucuronide hydrolyzed per minute per mg of protein and the results as represented as relative activity respect with to the wild type strain UW136. Protein was measured by the Lowry Method [36].

### *Western-blot* assays

Detection of RpoS levels in *A*. *vinelandii* cells grown for 36 hours in PY medium was carried out by western blot analysis as previously described [15]. For the determination of *in vivo* stability of RpoS, the strains were grown as mentioned above and protein synthesis was stopped by adding kanamycin (0.5 mg/mL). Relative protein levels of RpoS were estimated by densitometry analyses in the ImageJ software [37].

### *In silico* analysis

DNA and amino acid sequences of *relA*, *spoT*, *dksA*, and *rpoZ* from *A*. *vinelandii* were retrieved from KEGG. Nucleotide sequence alignments were done by NCBI-BLAST (Basic Local Alignment Search tool) using as a reference the *A*. *vinelandii* genome accession number NCBI ID 322710 (https://blast.ncbi.nlm.nih.gov/Blast.cgi). Conserved amino acid sequences were analyzed using the Clustal Omega program (https://www.ebi.ac.uk/Tools/msa/clustalo/).

### Quantitative real time PCR (RT-qPCR) and RNA-seq assay

For RT-qPCR experiments, total RNA was extracted from *A*. *vinelandii* strains grown in PY liquid medium at 36 hours and 30°C. The RNA samples were extracted with TRIzol^™^ Max^™^ Bacterial RNA Isolation Kit (Thermo Scientific) as a manufacturer protocol. The quality and quantity of the RNA were determined by Nanodrop One^C^ (Thermo Scientific). 2μg of RNA were employed to treat with DNAse (Thermo Scientific) following manufacturing instructions. The Revert Aid H Minus First Strand cDNA Synthesis kit from Thermo Scientific was used to synthesize cDNA using reverse oligonucleotides listed in S1 Table. A PCR was carried out for these cDNAs to confirm the correct synthesis. A LightCycler480 System (Roche Diagnostics) equipment and SyBR-Green were used for fluorescence quantification. A q-PCR assay corresponds to a 3 min preincubation at 95 C, 40 cycles at 60 C for 1 min with a 15 second interval between cycles. A *gyrA* gene was used as an internal control.

For RNA-seq analysis, an RNA extraction kit was employed (Total RNA Purification Kit, Jena Bioscience) according to manufacturer instructions. The analytical quality of RNA was determined using an Agilent Bioanalyzer 2100 system, before sequencing of ribosomal RNA-depleted RNA. The ribosomal depletion was carried out by RiboMinus rRNA depletion kit for bacteria (Thermo Fisher Scientific). Libraries were generated using TruSeq Stranded mRNA Sample Preparation Kit (Illumina). Sequencing was performed by NextSeq 500 (Illumina) with the kit NexSeq 500 High Output kit v2.5. The clean data was mapped to the reference genome of *A*. *vinelandii*

DJ from NCBI Genomes. The alignment was carried by Smalt software and the coverage was determined by CoverageBed from Bamtools software. The differential expression analysis was carried by IDEAMEX software (Integrative Differential Expression Analysis for Multiple EXperiments) that use four packages, edgeR, DESeq2, limma, and NOISeq. The conjunction of the genes by these four packages determines the real differential expression between UW136 and UW*dksA* mutant.

The significantly differentially expressed genes (DEGs) were determined as log_2_ FC ±1, p-value <0.05, and counter per million >1 (CPM>1) are reported in S1 File. The biological relevance of the significant DEGs was estimated by list enrichment analysis and the Kyoto Encyclopedia of Genes and Genomes (KEGG) [38]. The analysis of enrichment GO: terms were established at a log_2_ FC ±1 and FDR 0.05 [39, 40].

### PHB quantification

PHB was extracted from cells grown in PY liquid at 30°C and hydrolyzed with concentrated H_2_SO_2_ as described by Peña *et al* 1997 [41]. The crotonic acid produced by the PHB hydrolysis was measured using the method described by Law and Slepecky 1961 [42].

## Supporting information

S1 Fig The *rpoZ* and *spoT* genes are transcribed as a bicistronic operon. The non-polar mutation in UW*rpoZ* did not affect *spoT* transcription. (A) Physical map of the *A*. *vinelandii rpoZ* (Avin02820) and *spoT* (Avin02810) genes. The arrows indicate the oligonucleotides used to determine the transcript corresponding to the intergenic region of the *rpoZ-spoT* operon. (B) RT-PCR using total RNA from UW136 to amplify a 120 bp of the *rpoZ-spoT* intergenic region. Total DNA from UW136 was used as positive control. (C) Transcription of *spoT* in UW*rpoZ* compared with its parental UW136 strain, determined by RT-qPCR.
(TIF)

S2 Fig Physical map of the *rpoS* region including promoters P1, P2 and P3, and fragments used for the construction of the *rpoS-gusA* transcriptional fusions, and relative activity from P2 and P3 promoters in UW*dksA* strain. (**A)** The -10 and -35 regions for P1, P2, P3, promoters are indicated by small squares. The DNA fragments used for the construction of *rpoS*::gusA fusions are represented as rectangles. **(B)** and **(C)**. Relative -glucuronidase activity in UW136 and UW*dksA* strains carrying P2rpoS::gusA and P3rpoS::gusA fusions respectively.
(TIF)

S3 Fig The ribosomal proteins and some enzymes of the glycolysis and pyruvate synthesis are differentially expressed in the UW*dksA* strain. **A.** Expression profile of ribosomal proteins in UW*dksA*. **B.** Glycolysis and pyruvate pathways from *A*. *vinelandii* KEGG pathways, that showed differential expression in UW*dksA* respect to wild type.
(TIF)

S1 Table Oligonucleotides used in this study. (PDF)

S1 Raw images (PDF)

S1 File RNA-seq results. *Fold change* ±1 from UW*dksA* strain.
(TXT)
