# Supplementary figures and images for: The stringent response regulates the poly-β-hydroxybutyrate (PHB) synthesis in Azotobacter vinelandii
Source: PLoS One. 2024 Apr 4;19(4):e0299640. doi: 10.1371/journal.pone.0299640 (PMC10994330; doi:10.1371/journal.pone.0299640)

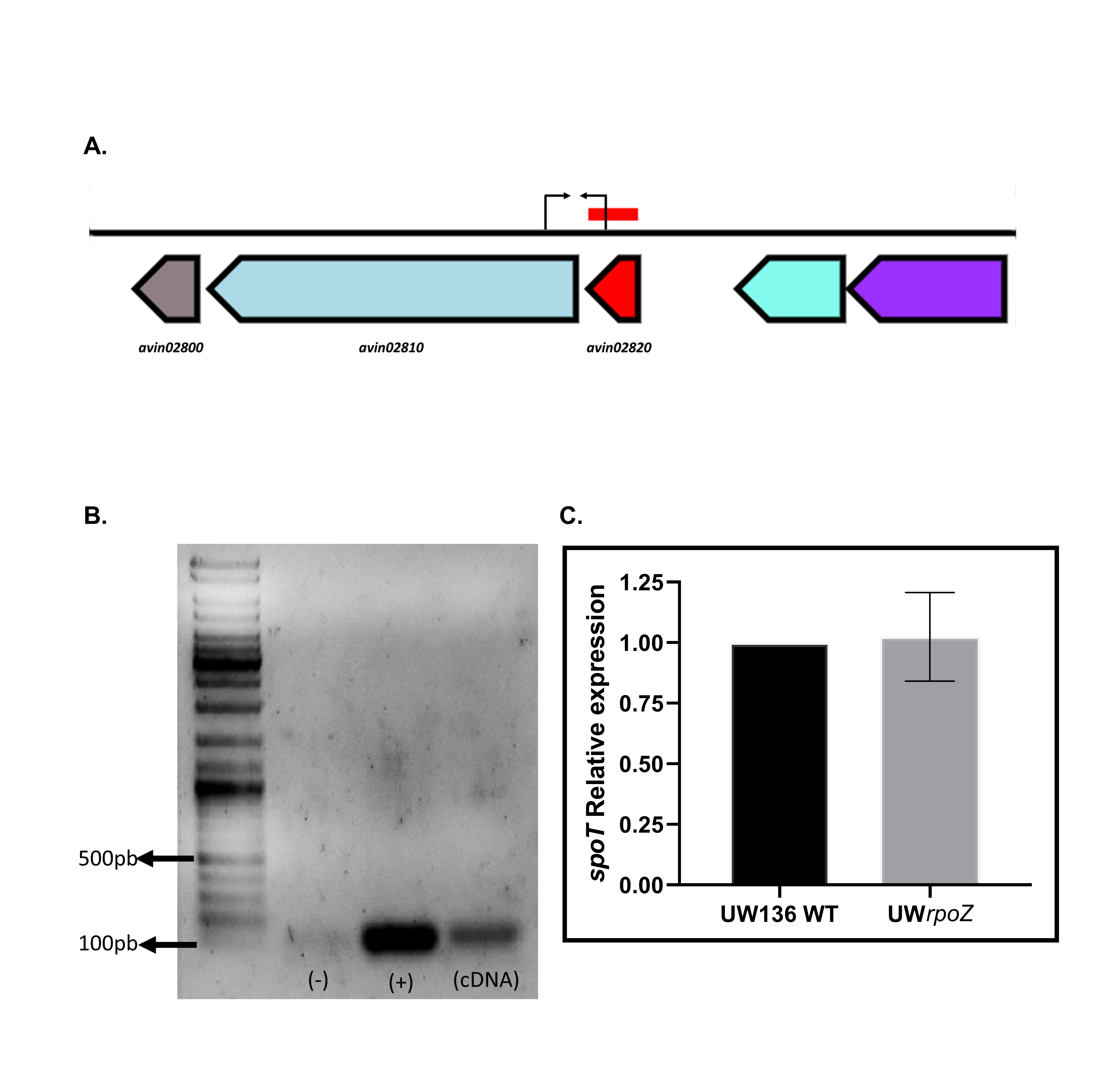

Supplement: S1 Fig — The non-polar mutation in UWrpoZ did not affect spoT transcription. (A) Physical map of the A. vinelandii rpoZ (Avin02820) and spoT (Avin02810) genes. The arrows indicate the oligonucleotides used to determine the transcript corresponding to the intergenic region of the rpoZ-spoT operon. (B) RT-PCR using total RNA from UW136 to amplify a 120 bp of the rpoZ-spoT intergenic region. Total DNA from UW136 was used as positive control. (C) Transcription of spoT in UWrpoZ compared with its parental UW136 strain, determined by RT-qPCR. (TIF) [file pone.0299640.s001.tif]

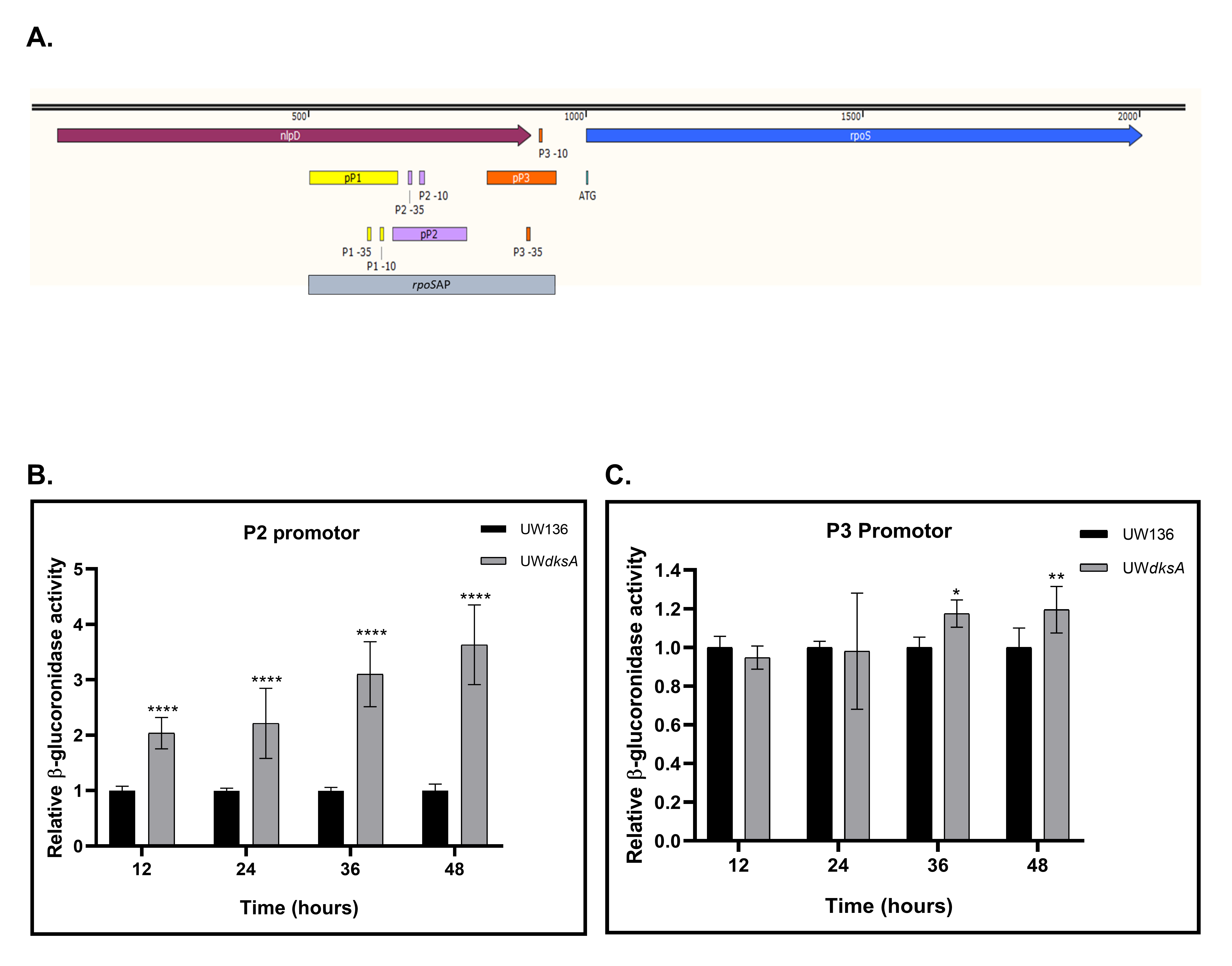

Supplement: S2 Fig — (A) The -10 and -35 regions for P1, P2, P3, promoters are indicated by small squares. The DNA fragments used for the construction of rpoS::gusA fusions are represented as rectangles. (B) and (C). Relative -glucuronidase activity in UW136 and UWdksA strains carrying P2rpoS::gusA and P3rpoS::gusA fusions respectively. (TIF) [file pone.0299640.s002.tif]

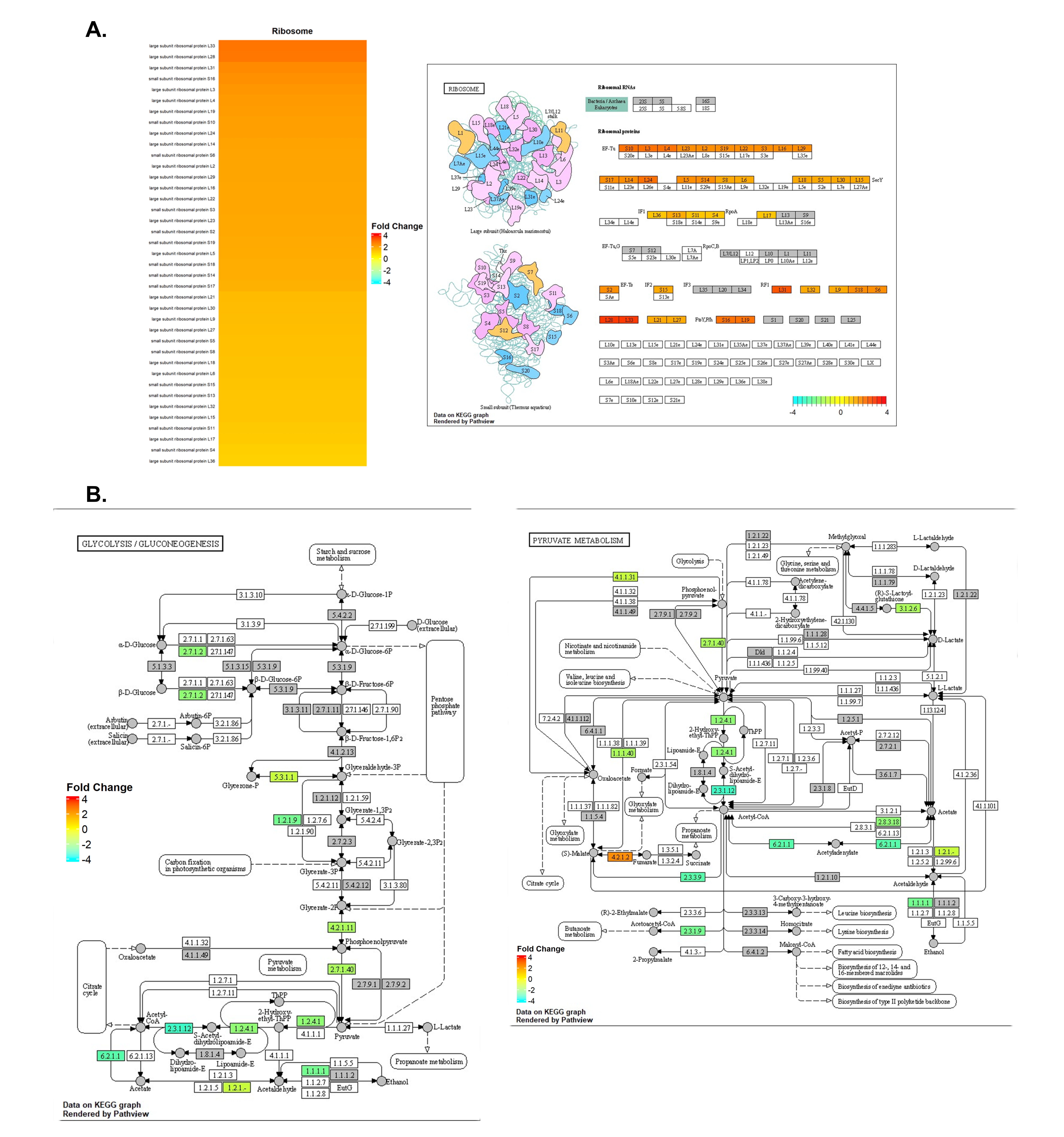

Supplement: S3 Fig — A. Expression profile of ribosomal proteins in UWdksA. B. Glycolysis and pyruvate pathways from A. vinelandii KEGG pathways, that showed differential expression in UWdksA respect to wild type. (TIF) [file pone.0299640.s003.tif]
