# Supplementary material for: The stringent response regulates the poly-β-hydroxybutyrate (PHB) synthesis in Azotobacter vinelandii
Source: PLoS One. 2024 Apr 4;19(4):e0299640. doi: 10.1371/journal.pone.0299640 (PMC10994330; doi:10.1371/journal.pone.0299640)
Supplement: S1 Table — (PDF) [file pone.0299640.s004.pdf]

Oligonucleotides		
spoT-Fw	GGTCGACTACAACGTCATC	This study
spoT- Rv	GCCTTGTCGGTATTGATCAC	This study
relA-Fw	CCCGTAATGGACTGAACAAC	This study
relA-Rv	ACGATTGCCCACTATAGGAC	This study
dksA-Fw	CTG TAG GGC ATG GGC CTT CC	This study
dksA-Rv	CGC ACG CAG GAT CAA CTG C	This study
rpoZ-Fw	ACGGCCATTTGGCCCTTC	This study
rpoZ-Rv	ATGCTGGCCAAGGACGCTC	This study
dksAC-Fw	GGATCCCGCGCGTGATCA	This study
dksAC-Rv	TCTAGAGCAGGATCAACTGCCGAT	This study
relAC-Fw XbaI	GGATCCATGGTACAGGTGAGAGCG	This study
relAC-Rv BamHI	TCTAGATCTAGACGGCGTCAGGAAACC G	This study
rpoS P1 Fw	TCTAGAGTGCACAGCCGGCGGTGT	This study
rpoS P1 Rv	GAATTCACCGACCCATCAGACGCAG	This study
rpoS P2 Fw	TCTAGAGATGGGTCGGTTGTGTAC	This study
rpoS P2 Rv	GAATTCTTCACCTGTTGTCCTTCC	This study
rpoS P3 Fw	TCTAGAACCGATCGGGTGAAGC	This study
rpoS P3 Rv	GAATTCTTGGACACCCGAGCCCTTA	This study
rpoS-Fw BamHI	GGATCCGCTCTCAATATAAAAGCAAAA GAAG	This study
rpoS-Rv XbaI	TCTAGACATCTTTTTCACTGGAACAGC	This study
qRT-rpoS Up	AGG ATG TCC TGG ACG ATG AG	This study
qRT-rpos Down	TCC AGC GCC CTA GTG TAG TC	This study
qRT-gyrA Up	CCAGCAAGGGCAAGGTCTA	This study
qRT-gyrA Down	TCGTCCAGCGGCAACAGGT	This study
qRT-phbR Fw	CCACATGCGTTTGGTTTCTTG	This study
qRT-phbR RV	TCTGCCCTAAGCGTCTCCTC	This study
qRT-phbB Fw	TCCGCAATCCAAACGCAAAG	This study
qRT-phbB Rv	GTGCTCTCCGAACGGTTAC	This study
qRT-avin27210 Fw	CGGATAGCTTTCCGTTCAACGG	This study
qRT-avin27210 Rv	ATCAGCCGCTTGTTGTCGT	This study
qRT-eno Fw	AGGACACCACCATCGCCG	This study
qRT-eno Rv	TTGCCCTGCAAGGCCTCC	This study
qRT-avin27240 Fw	ATGCTGCGTAGAACCAAGATC	This study
qRT-avin27240 Rv	TGGGAGAAGTTCAGGCGCA	This study
qRT-avin27250 Fw	CCG TGC TCG ACG AAA GCA	This study
qRT-avin27250 Rv	GCA CCG GGC AGT TCA TCC	This study
qRT-avin34530 Fw	CCA TCA TCT GGG AGG GCG AC	This study
qRT-avin34530 Rv	CGC GAT GCA CGT CCT GAC	This study
qRT-avin39300 Fw	CCG CAG ACG CAT TGG CC	This study

qRT-avin39300 Rv	GCC TCG TAC TTG CGG ATC AG	This study
qRT-avin41750 Fw	GAG ATC GAC GCC GTG GTG	This study
qRT-avin41750 Rv	GAG GAA GCG GAT GAC GCG G	This study
qRT-avin46160 Fw	CCG TTG ATG AAC ACC GCC G	This study
qRT-avin46160 Rv	TCC AGA CTG TGC GAG TGC TG	This study
qRT-avin46170 Fw	GTA CAT CGG CCA GGA GGC G	This study
qRT-avin46170 Rv	ATC TCG GCC ACG ATC GCG	This study
