# Supplementary material for: The stringent response regulates the poly-β-hydroxybutyrate (PHB) synthesis in Azotobacter vinelandii
Source: PLoS One. 2024 Apr 4;19(4):e0299640. doi: 10.1371/journal.pone.0299640 (PMC10994330; doi:10.1371/journal.pone.0299640)

Original gels to Fig.3A

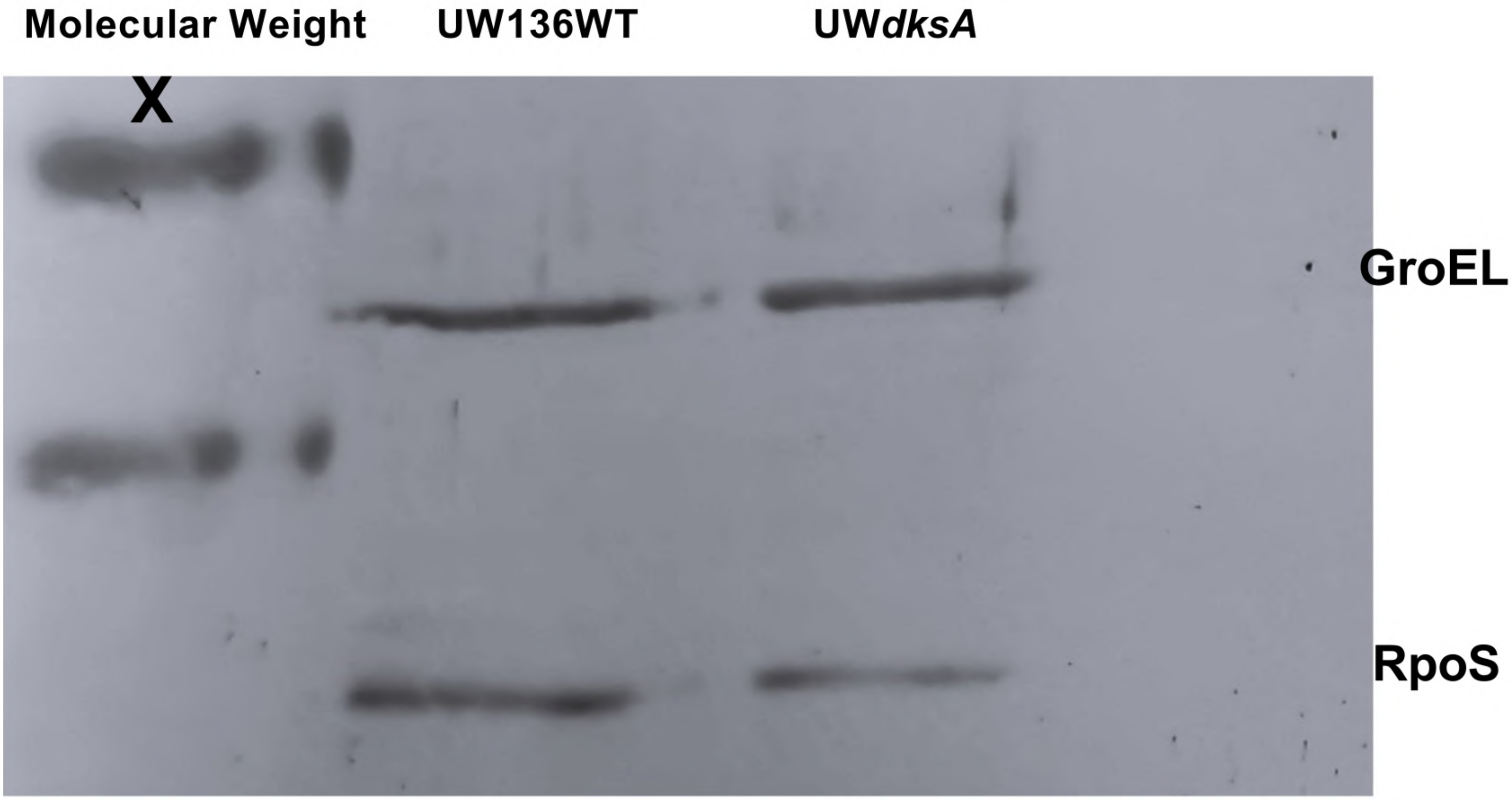

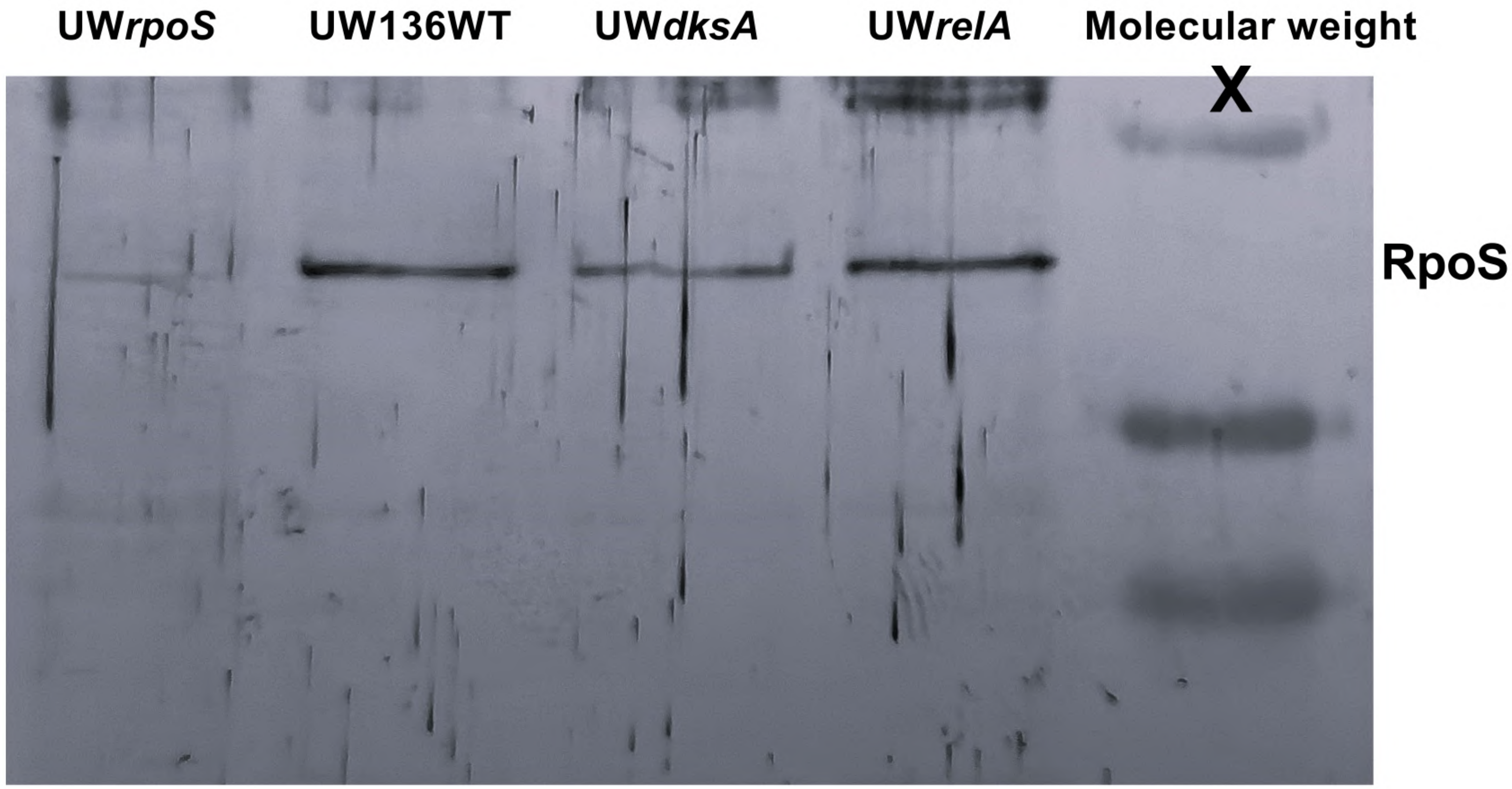

Original gels to Fig.3B

UW136 WT

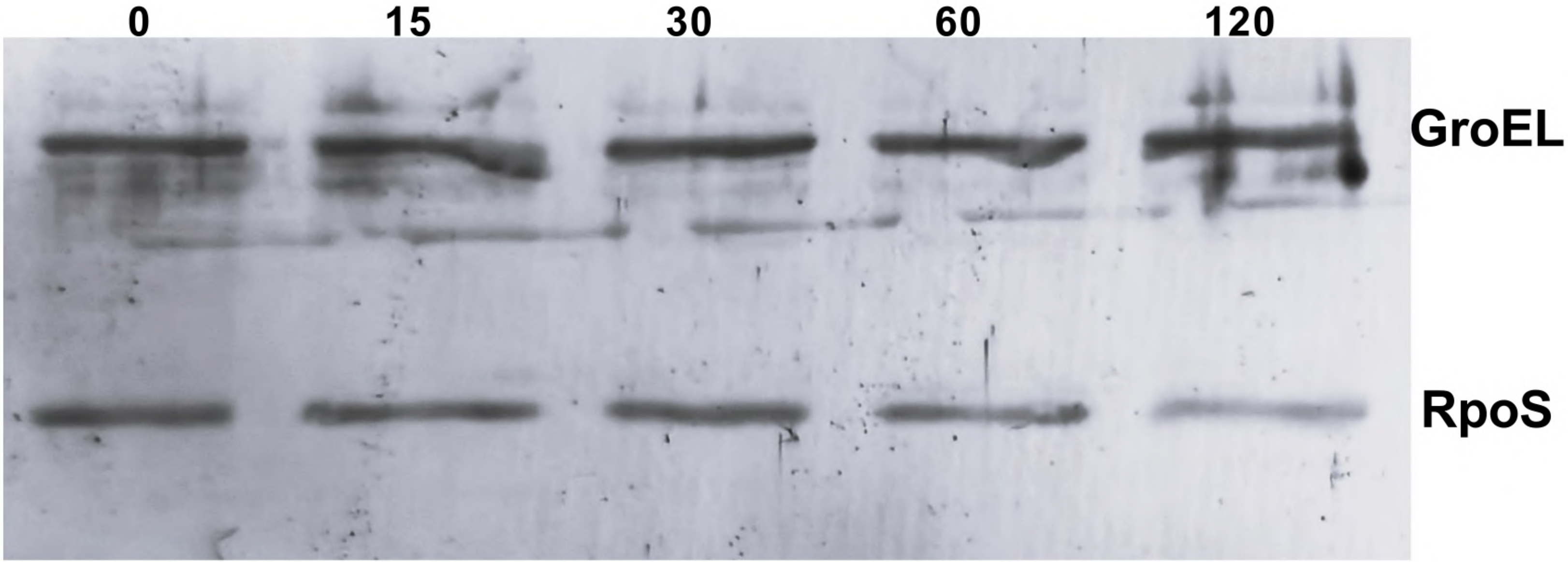

Original gels to Fig.3B

UW*dksA*

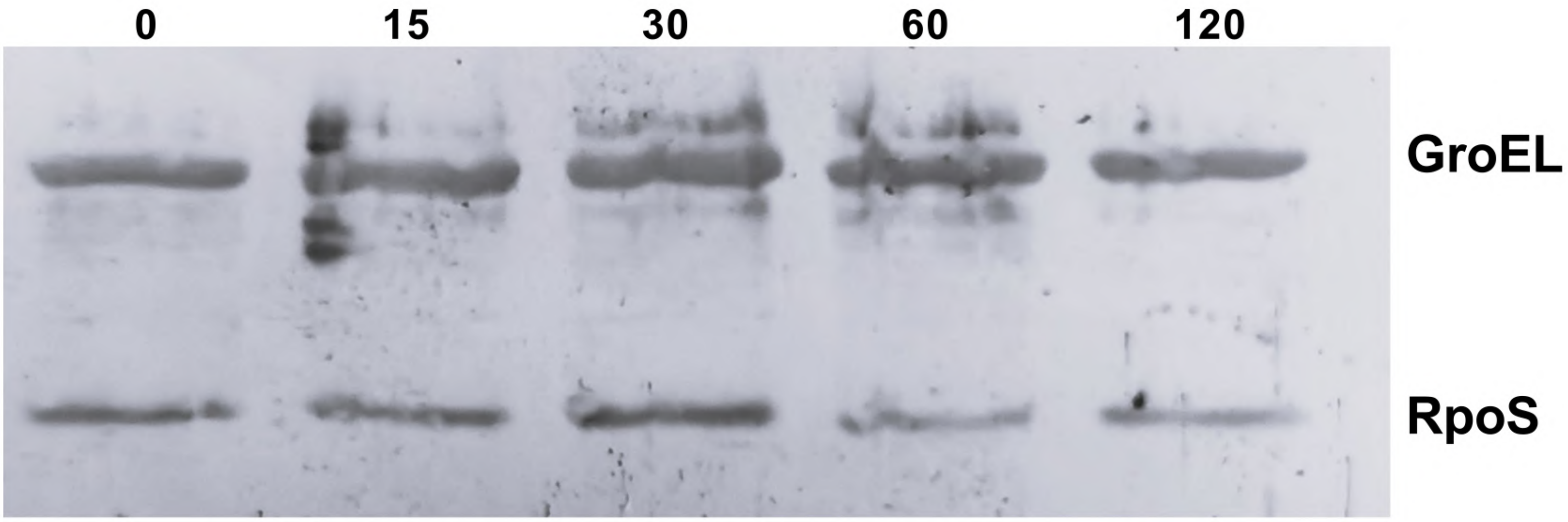

UW*ptsP*

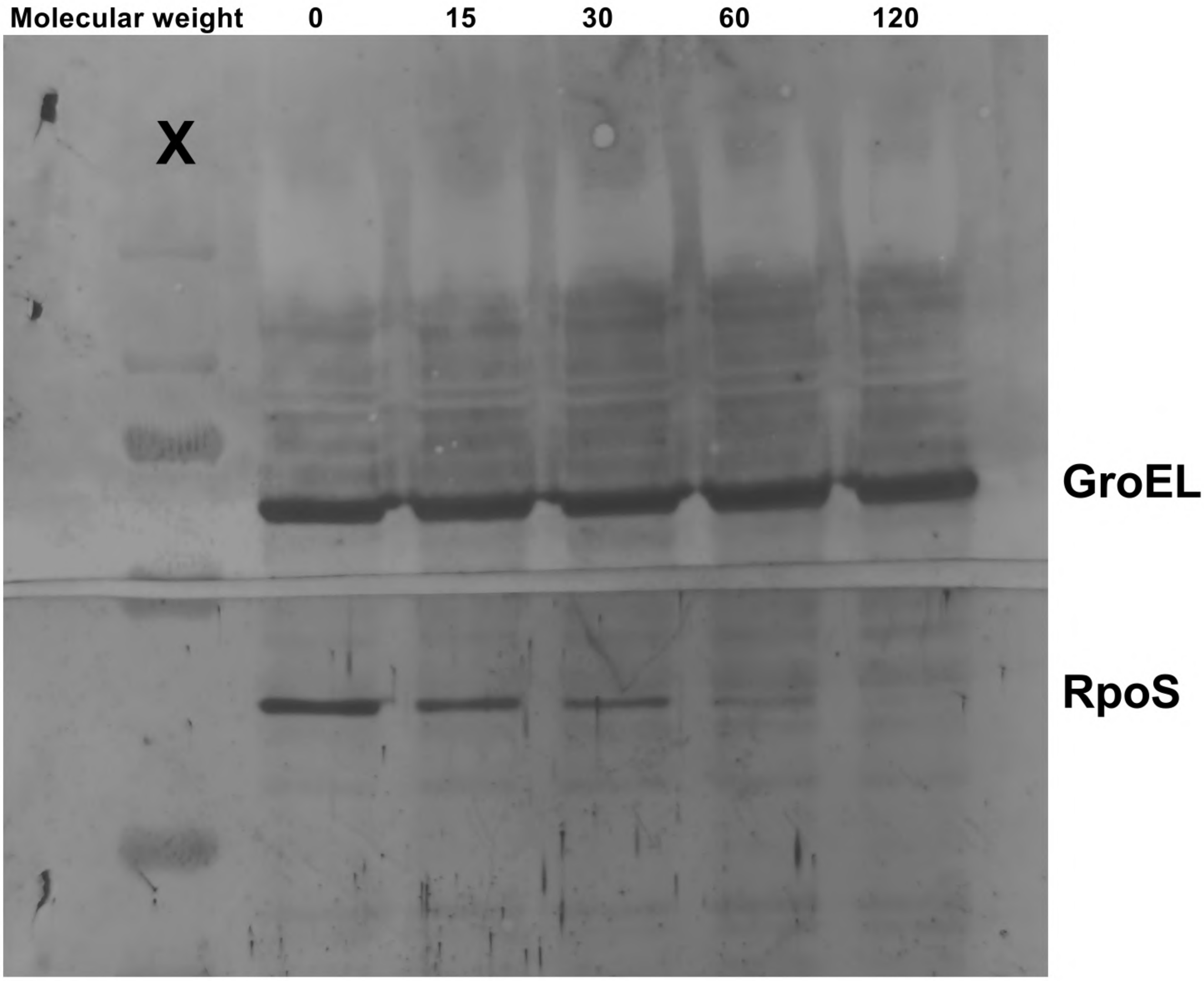

Original gel to Fig.4C

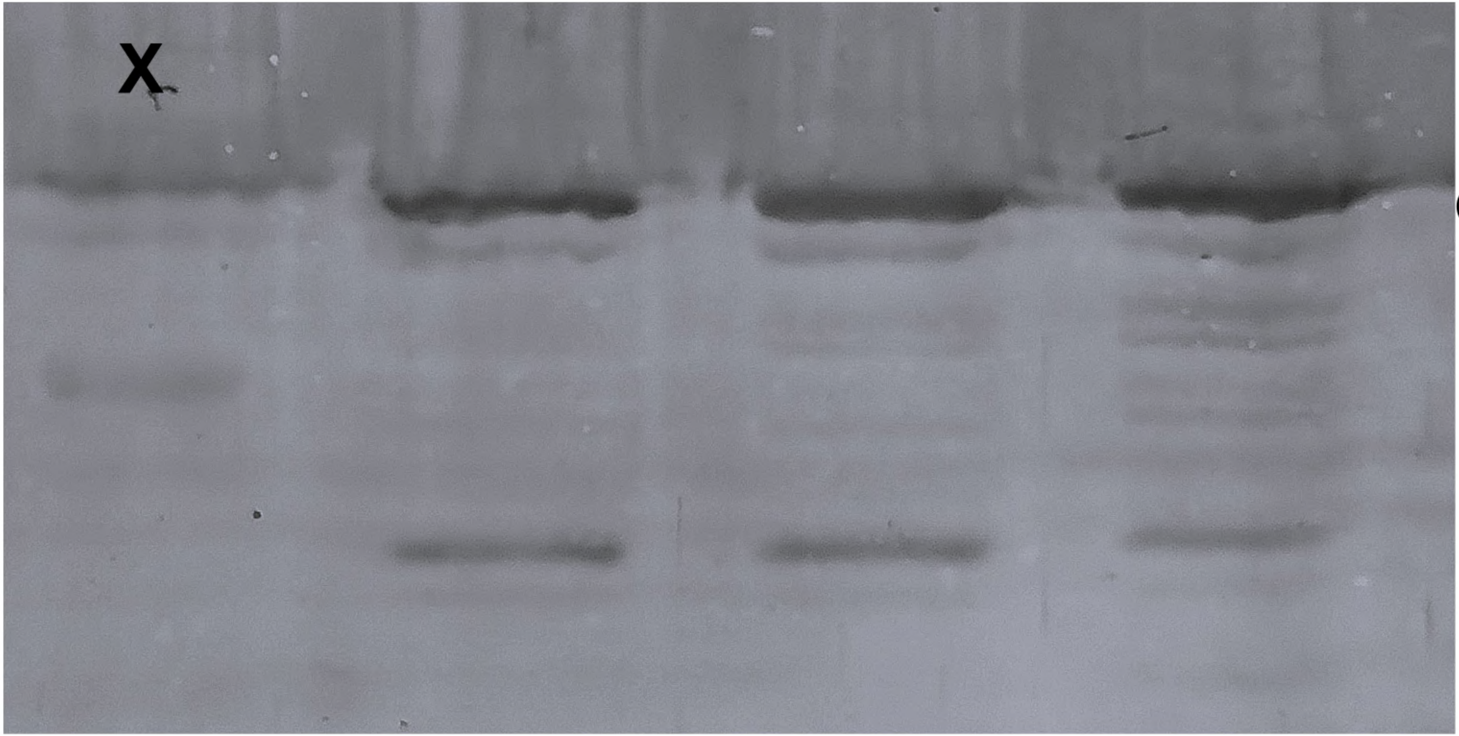

Supplement: S1 Raw images — (PDF) [file pone.0299640.s005.pdf]
